# Do Academic Libraries Contribute to Students’ and Communities’ Wellbeing?: A Scoping Review

**DOI:** 10.3390/healthcare13020179

**Published:** 2025-01-17

**Authors:** Shivani Suresh, David Lim, Kanchana Ekanayake, Amit Arora

**Affiliations:** 1 School of Health Sciences, Western Sydney University, Locked Bag 1797, Penrith, NSW 2751, Australia; a.arora@westernsydney.edu.au; 2 Library Services, Western Sydney University, Penrith, NSW 1727, Australia; 3 Translational Health Research Institute, Western Sydney University, Campbelltown, NSW 2560, Australia; david.lim@uts.edu.au; 4 Improving Palliative, Aged and Chronic Care through Clinical Research and Translation, Faculty of Health, University of Technology Sydney (UTS), Broadway, NSW 2007, Australia; 5 University of Sydney Library, The University of Sydney, Camperdown, NSW 2006, Australia; kanchana.ekanayake@sydney.edu.au; 6 Discipline of Child and Adolescent Health, The Children’s Hospital at Westmead Clinical School, Faculty of Medicine and Health, The University of Sydney, Westmead, NSW 2145, Australia; 7 Health Equity Laboratory, Campbelltown, NSW 2560, Australia; 8 Oral Health Services, Sydney Local Health District and Sydney Dental Hospital, NSW Health, Surry Hills, NSW 2010, Australia

**Keywords:** wellbeing, health promotion, library services, universities, students, community

## Abstract

**Background/Objectives**: Academic libraries offer a range of activities and initiatives for their students and community users. However, wellbeing, as a concept in academic libraries, is not very well defined and is poorly understood. The objective of this scoping review was to examine the role of academic libraries in student and community wellbeing, identify the various kinds of activities and initiatives that they carry out to address their wellbeing, and uncover gaps that might require further research. **Methods**: An extensive search was conducted in the Library Information Sciences Association (LISA), Education Resources Information Centre (ERIC), Medline (OVID), Scopus, and Web of Science (WOS) databases. Grey literature was searched on a selection of library websites and digital repositories. Data were extracted from studies that met the inclusion criteria for the scoping review. Themes were identified by the authors and reported as a narrative summary. **Results**: Of the 5437 records identified, a total of 40 documents were included in this scoping review. The authors identified 11 different kinds of activities and initiatives carried out in academic libraries that promote student and community wellbeing, i.e., (1) animal-assisted activities; (2) facilitating dialogues about belonging and identity; (3) fun recreational activities; (4) study support; (5) physical activity promotion; (6) meditation, yoga, and mindfulness; (7) book clubs; (8) art exhibitions; (9) technology and digital support; (10) free food and tea; and (11) health awareness. These activities were found to promote the physical, emotional, and social wellbeing of student and community users. **Conclusions**: This review highlights the need for further systematic research on the long-term effects of wellbeing initiatives and activities on both student and community users, and how they might impact aspects of wellbeing for specific population groups such as senior citizens and LGBTQIA members. This scoping review demonstrates the potential of academic libraries in promoting health and wellbeing for their users.

## 1. Introduction

For decades, the discipline of public health has recognised that social determinants of health have the greatest impact on overall quality of life and wellbeing compared to individual approaches to healthcare [1]. Wellbeing, often used interchangeably with health, embodies a dual and broader definition in public health: “encompassing quality of life and the ability of people and societies to contribute to the world with meaning and purpose” and “the positive state experienced by individuals and societies” [2]. Understandings of wellbeing and health can vary across disciplines, cultures, individuals, and time, reflecting its expansive and ever-changing nature as a construct. Together, physical, mental, and social wellbeing, sometimes also referred to as the “Health Triangle” by researchers, offers a way to see connections between health and wellbeing [3].

Wellbeing is multidimensional; it can be assessed objectively in terms of GDP by measuring six dimensions (health, job opportunities, socio-economic development, environment, safety, and politics) and subjectively through self-reports on five dimensions (the role of human genes, universal needs, social environment, economic environment, and political environment) [4]. To actualise a sense of wellbeing, one must be equipped with tools, awareness, and an environment to lead a life of purpose, meaning, satisfaction, and pleasure [5]. In practice, challenges in attaining wellbeing through social determinants of health exist and can only be addressed with the help of a wide range of stakeholders that go beyond the health sector.

Debates and deliberations regarding health promotion have existed within public librarianship for a long time [6]. The public library, as a “democratic equaliser”, is often portrayed as a robust community centre that helps people care for their individual and collective lives (p. 191) [7]. In the past, libraries have acted as critical nodes of disaster response, as community spaces for accessing health-related information such as family planning information, and as a hub for social and health workers [6]. Even prior to the COVID-19 pandemic, public libraries have stepped in to support the emotional and mental health of the communities that they serve [6]. For example, public librarians often support the literacy needs in their communities by organising English as a second language and digital literacy programs [7]. Libraries foster social support and cohesion by promoting civic engagement in social justice issues [8]. They mitigate social exclusion by serving as “safe spaces” for vulnerable groups [8,9]. As physical spaces, libraries are often designed to relieve stress [8], for which they are often described as “community assets” [10]. As public libraries, academic libraries are both massive opportunities and gaps in this regard.

Academic libraries (university, college, higher education, or tertiary library), as a public libraries, offer an interesting opportunity to study wellbeing because of their open nature, physical presence, and attitudinal footprint [11]. A mixed picture emerges when one thinks of users of an academic library. Users of an academic library generally and broadly include students and the broader community [12]. Although academic libraries are not usually formally featured in universities’ wellness strategies, their programs significantly contribute to the wellbeing of their users, particularly students [13].

University students have some of the worst wellbeing indicators in comparison to any other population group. A survey conducted in 19 universities across eight countries found that of the 13,984 respondents, 31% screened positive for at least one mental health disorder [14]. A key finding in another recent survey of 56,375 university students at an Australian university revealed that at least one-third of the participating students experienced psychological distress, much higher than the figures that exist for the general population [15]. In recent years, universities have seen an interesting shift in thinking about wellness and wellbeing, from a traditional focus on problem behaviours such as drinking, suicide, and sexual violence amongst its students to thinking about holistic health and wellbeing [13]. With the range of initiatives already on offer for both students and communities (through library outreach services), academic libraries are well positioned to be a driving force in improving wellbeing outcomes for our society.

Academic libraries do more than serve as data depositories and quiet spaces. They are places of immense potential when it comes to improving health and wellbeing for their students and community users. However, connections between academic libraries and wellbeing are somewhat tenuous and unclear. Firstly, there is a gap in the literature regarding how wellbeing is conceptualised within academic libraries, in that the types of available evidence in the field are yet to be identified, there is a lack of key concepts and definitions, and key characteristics or factors related to the concept of wellbeing are yet to be identified. Secondly, to date, there is no review that examines the role and scope of academic libraries in student and community wellbeing.

In light of the evidence on the role that academic libraries play for both students and community users, academic libraries could have an important impact on their overall wellbeing. However, there has been no scoping review undertaken focusing on their wellbeing initiatives and their potential role in health promotion. Scoping reviews have become an increasingly useful tool amongst researchers [16]. They map and present the breadth of literature and shed light on the research gaps, while leaving detailed analysis of concepts, theories, and topics to other forms of analytical research [17]. The authors believe that a scoping review would be invaluable in identifying wellbeing initiatives and activities and showcasing the ways in which academic libraries improve student and community wellbeing.

### Review Questions

This review aims to answer the following research questions:

Do academic libraries contribute to their users’ (student and community) wellbeing? If so, how?
(a) What are the different kinds of wellbeing initiatives and activities that academic libraries offer?
  (b) How do these contribute to wellbeing of its student and community users?
What are the gaps in the literature around the role of academic libraries’ contribution to user wellbeing?

## 2. Methods

### 2.1. Study Design

A scoping review framework outlined by Arksey and O’Malley [18] was selected for this study. A scoping review helps researchers to map an area of interest; examine the nature, range, and extent of literature; present and disseminate research findings; and identify the research gaps in the literature. A scoping review was best suited for undertaking this review to gather a diverse body of evidence (case studies, opinion pieces, quantitative research, qualitative research) on this emerging research topic. This study was conducted using a JBI scoping review methodology [19] and reported according to the PRISMA extension for Scoping Reviews (PRISMA-ScR) guidelines [20]. The protocol of this scoping review was registered on Open Science Framework “https://osf.io/72fhq (accessed on 14 January 2025)”.

### 2.2. Search Strategy

The following databases were searched to gather data: Library Information Sciences Association (LISA), Education Resources Information Centre (ERIC), MEDLINE (Ovid), Scopus, and Web of Science (WOS). The search for literature was conducted from 29 November 2023 to 4 December 2023. The search strategy, including the keywords and subject headings, was adapted for each database to capture all the literature relevant to the topic, and no limits were used for the search. Wherever necessary, forward and backward citation tracking was used to identify resources in consultation with a school librarian, and the search strategy was subsequently refined. Boolean Operators (‘AND’ and ‘OR’), truncation, and phrase searching were used where appropriate to manage the search outputs. The search strings were verified using the PRESS, i.e., Peer Review of Electronic Search Strategies, checklist [21]. The search strategy was pre-tested in the MEDLINE (OVID) database and subsequently adapted for other databases. In addition to the database searches, a grey literature search was conducted on Google and Google Scholar advanced search using a simplified search input that captured all three dimensions of the topic “academic libraries user wellbeing”, and the first 5 pages of results were included. In addition, the authors hand-searched grey literature using the same simplified search on library websites—International Federation of Library Associations and Institutions (IFLA), Council for Australian University Libraries (CAUL), American Library Association (ALA), and Australian Library and Information Association (ALIA). Search strings are attached in the Supplementary Material section (Supplementary Material S1: Search Strategy).

### 2.3. Inclusion Criteria

The Population, Concept, and Context (PCC) framework guided the inclusion criteria for this scoping review.

- Population: Students and Community Users of the Academic Library

This review considered studies where the population of interest is students and community users of the academic libraries. Student users were defined to include undergraduate, postgraduate, or working professionals who are affiliated with an academic library as students. Community users were defined as residents of the region who live in the vicinity of an academic library or are external users of an academic library.

- Concept: Wellbeing in the Academic Library

Records that discussed wellbeing initiatives focused on maintaining or improving the existing health status of student or community users within an academic library were included in this review. This included records that described activities that are organised by the library, sector-wide comprehensive reviews of initiatives in academic libraries, or studies that detailed survey findings with the intent to improve wellbeing for its users. For this scoping review, wellbeing was not pre-defined, and all aspects of wellbeing were considered.

- Context: Academic Libraries Globally

This review considered studies that were situated in an academic library of a university or college or tertiary education context, regardless of their geographical location.

To be comprehensive, we did not apply a date range to the search, but for practical reasons associated with language proficiency, we only screened for records available in English. No other exclusion criterion was applied.

### 2.4. Study Selection and Data Extraction

Studies identified through the electronic databases, grey literature, and citation chasing were uploaded into Covidence for removing duplicates, screening, and selection [22]. Two reviewers (SS and AA) independently assessed the titles and abstracts of the search results to determine whether they met the inclusion criteria as per Pollock et al. (2021) [23]. All citations that met the inclusion criteria were retrieved in full-text form. Any disagreements were resolved through discussion amongst all co-authors. For example, if SS and AA disagreed on whether a paper met the inclusion criteria, DL would be consulted, and a joint decision would be reached based on consensus. The reasons for excluding studies after reading full-text articles are reported in Supplementary Material S3. The process of study selection was carried out in accordance with the PRISMA-ScR checklist (Supplementary Material S4) and presented as a flow diagram.

A standardised data extraction form was developed and pilot-tested by the review team. The following information was extracted: study title, first author, year of publication, study design, institution/university, location, target population, aim/purpose, initiative/activity description, key findings, and gaps in the literature. A thematic synthesis approach was utilised by the review team to report the review findings.

## 3. Results

### 3.1. Search Results

**Figure 1:**
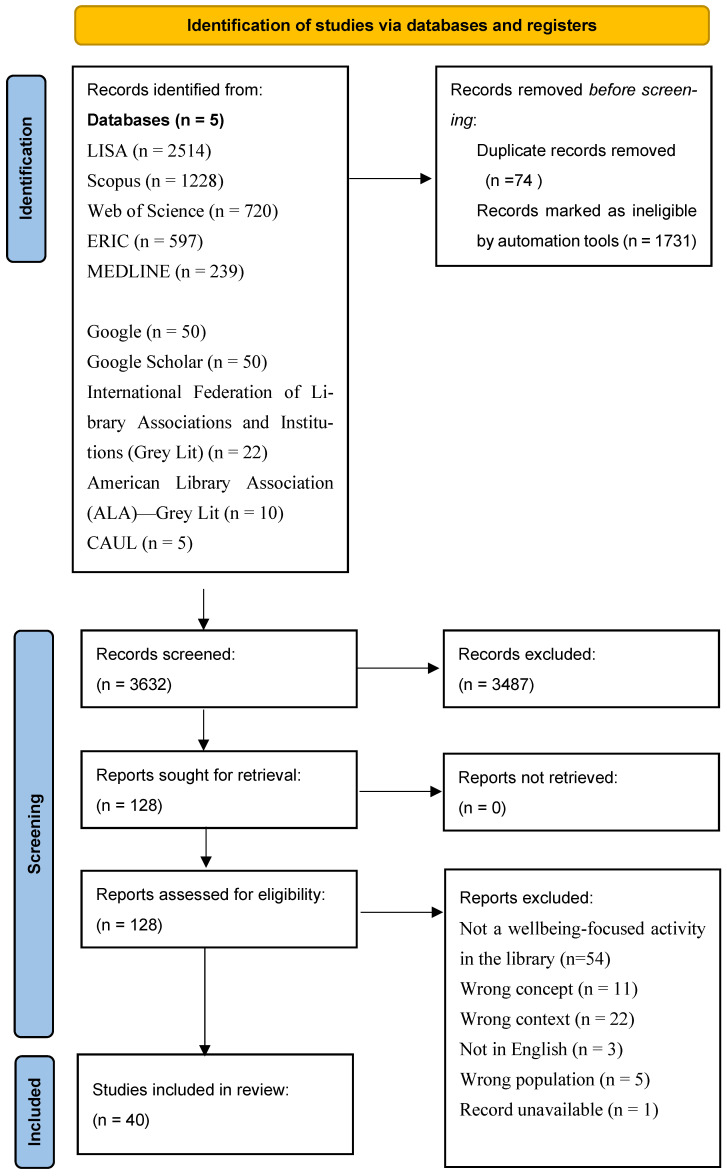
PRISMA Flowchart [24]. Characteristics of included studies.

Of the 5437 citations retrieved, 1805 were duplicates and were removed. The remaining 3632 citations were screened by title and abstract. Of the 128 citations that progressed to full-text screening, 40 citations were included, as shown in the PRISMA-ScR flowchart shown in Figure 1.

#### 3.1.1. Distribution by Year of Publication

The included results were published between 1983 and 2023. The highest number of publications (n = 10; 25%) was in 2019.

#### 3.1.2. Distribution by Country

The majority of the publications were from the USA (n = 27; 67.5%), followed by the United Kingdom (n = 7), Canada (n = 3), Ghana (n = 1), and South-East Asia, i.e., Brunei, Darussalam, Indonesia, Thailand, Laos, Myanmar, Malaysia, Cambodia, the Philippines, Vietnam, and Singapore (n = 1).

#### 3.1.3. Distribution by Study Design

The majority of the citations were case studies (n = 12; 30%) and text and opinion pieces (n = 12; 30%). Other kinds of study types included survey (n = 8), conceptual paper (n = 1; 2.5%), quasi-experimental design (n = 1; 2.5%), literature review (n = 2; 5%), non-randomised experimental study (n = 1; 2.5%), empirical research (n = 1; 2.5%), and qualitative study (n = 2; 5%).

#### 3.1.4. Distribution by Population Subgroup

A total of 34 publications reported on wellbeing initiatives relevant to students, and only 6 publications related to community. Several specific subgroups of student users were identified from the results. Within the results that focused on student users, most of the wellbeing initiatives in an academic library were targeted at students in general (n = 30). Other wellbeing initiatives were targeted to specific student cohorts such as students with disability (n = 1), health science and/or medical students (n = 2), international students (n = 1), and students who identify as LGBTQIA (n = 2). In total, only six results dealt with community users, of which only one result was focused solely on community, targeting senior residents. In the remaining five results, the activities and initiatives were organised for both students and the community.

### 3.2. Review Findings

- Do academic libraries contribute to their users’ (student and community) wellbeing?

All 40 citations in this review described activities and initiatives in academic libraries that indicated the same.

- What are the various kinds of wellbeing initiatives and activities within academic libraries?

Eleven different category types of activities/initiatives were apparent from the extraction of results. They are tabulated and summarised below (Table 1).

#### 3.2.1. Physical Wellbeing

Students: Across the various initiatives, five studies dealt with physical activity promotion. While most students reported being grateful to the library for taking active steps to help students with sedentarism through the introduction of bike and treadmill desks, there were other students who felt that it was a confusing experience. For instance, in Clement et al.’s (2018) paper, one student notes—“*I learn more effectively in a stationary position without sweat beads falling on my laptop or paper*” (p. 172) [53]. Although activities like yoga encouraged physical activity, students did not report the same sorts of confusion. Other activities that encouraged physical wellbeing included health awareness sessions about HIV, the provision of free food for students, and fun recreational activities like mini golf [56].

Community: Two studies explored the concept of physical wellbeing for community users. Norton’s (2019) study described an initiative targeted at disease prevention for community users, namely the Creative Campaign for HIV awareness organised by the University of Florida [45]. The project engaged the community through social media, generating approximately 297 clicks. However, an evaluation of how the initiative had impacted health behaviours was recognised as a limitation of the study. The survey findings in Peñaflor’s (2021) study also showcase various initiatives such as the Fit and Firm initiative and dance lessons that promote physical activity amongst the community [32].

#### 3.2.2. Mental Wellbeing

Students: Mental wellbeing was another aspect of wellbeing that appeared in the different categories of initiatives run by libraries. Art exhibitions in the library helped students feel seen and heard and encouraged coping with complex emotions like shame [49]. Meditation, yoga, and mindfulness activities and intentionally organised mindfulness spaces in the library were also appreciated and largely successful, especially when organised during exam week. Eleven citations reported on meditation, yoga, and mindfulness which help students cope with stress emotionally and psychologically when the semester is in session. Students reported that these activities helped them deal with stress and anxiety. As one student in Casucci’s (2019) paper reflects on the initiative, “It (yoga)…rejuvenates me” (p. 82) [26]. Health awareness campaigns that focussed on suicide prevention and mental health awareness were also received well [32]. Similarly, recreational activities run in the library were seen to improve the mood of students. Animal-assisted activities such as therapy dogs were found to significantly improve the emotional wellbeing states of students, particularly when run during the exam period [40]. For instance, participants in Edward’s (2022) paper reported an increase in self-reported levels of happiness [36].

An aspect of mental health that appears to be a recurring theme is cognitive wellbeing. With the move of academic libraries from being seen as information commons to being seen as learning commons in recent years, there appears to be an increased expectation that they provide support for students during their studies. Study support activities for students during finals week encouraged feelings of achievement and success amongst students [58]. Similarly, librarians provided technology and digital support to students, equipping them with skills that position them well for success [27].

Community: Thomas’s (2019) paper described a mental health initiative that encouraged people to talk about their problems through the medium of art [49]. The webpage linked to the initiative reported 1988 visits in a span of three years. Peñaflor’s (2021) study described a suicide prevention campaign in an academic library that raised awareness about mental health issues amongst community members [32].

#### 3.2.3. Social Wellbeing

Students: Across the different categories of initiatives, the social wellbeing of student users was addressed in 18 citations in four categories (namely book clubs, facilitating dialogues about identity and belonging, art exhibitions, and technology and digital support). For instance, in book club initiatives, international students, who tend to struggle to adjust to a new environment, find a sense of belonging when invited into spaces where they can have conversations about their experiences [44]. Similarly, art exhibitions that highlight LGBTQIA issues have been found to increase awareness and visibility for these groups, thereby aiding social cohesion and participation [48,60].

Community: Academic libraries provided vulnerable members of the community opportunities to improve their education levels and health outcomes. For example, the University of Louisville provided a computing skills class for the homeless [62]. Some Ghanaian academic libraries also provided maternal health information which helped in the process of attaining SDG 3 Goals [50]. Another initiative in Louisiana, the Senior CHAT (Consumer Health Awareness Training) program, provided senior citizens with basic computer instruction and encouraged them to seek information that related to their own health [52]. Seymour’s (2012) art exhibition initiative reportedly also promoted acceptance amongst community members regarding LGBTQIA issues [60].

- What are the gaps in the literature that require further research?

One of the main gaps is that most of the citations were texts and opinions (n = 12/40; 30%). There appears to be a lack of primary empirical research on how health and wellbeing are conceived and defined in these initiatives. Most of the citations do not quantitatively measure the impact of the wellbeing initiatives. Moreover, there was no longer-term or longitudinal research conducted in relation to these wellbeing activities, for example, on how the short-term impact may be sustained. Measuring and understanding impact are necessary to help understand the effectiveness of wellbeing initiatives in an academic library to improve wellbeing. The second major gap in our scoping review is the lack of specificity regarding who the target group is. To foster a sense of belonging and promote engagement, initiatives that address the unique challenges of different population groups may need to be considered. Only seven out of forty citations provide activities that are catered to specific groups such as LGBTQIA, migrant users, senior residents, and those with disabilities. Wellbeing for other minority groups, for example, those with learning difficulties, has not been explored. There is also a lack of specificity regarding what aspect of wellbeing the initiative is targeting. For instance, there were only six citations that were categorised as health awareness and five for physical health promotion. All other citations described activities or initiatives that were organised to support general wellbeing. Thirdly, there are only 6 out of 40 citations that target wellbeing activities towards the community. Apart from the aforementioned gaps, negative reporting on whether academic libraries contributed to wellbeing was scarce.

## 4. Discussion

### 4.1. Main Findings

This review sought to explore whether academic libraries contribute to user wellbeing and present the range of different initiatives that academic libraries offer to address wellbeing. To the best of the authors’ understanding, this is the first scoping review that offers a descriptive analysis focussing on both student- and community-user wellbeing in academic libraries. The findings in this review reveal that research in this area is fairly recent and predominantly located in Western countries such as the USA and UK, spanning forty years from 1983 to 2023. While it is beyond the scope of this review to explore the impact and efficacy of the wellbeing initiatives in libraries, it presented a broad overview of the wide-ranging efforts of academic libraries towards promoting wellbeing. To this end, the authors identified 11 categories of initiatives and activities (see Table 1) that academic libraries deliver for the wellbeing of both their student and community users. This review also highlighted that intentionally organised academic library spaces allow complex, difficult conversations about issues to take place all while ensuring safety.

From this review, it is also evident that most of the wellbeing initiatives in this review primarily cater to students. Amongst the 11 categories of initiatives identified, it is evident that academic libraries are employing various innovative approaches to promote wellbeing of their students, from light-hearted activities like animal-assisted activities to more serious initiatives like facilitating dialogues about identity and belonging. On the other hand, wellbeing initiatives for community users are less common in academic libraries. This may be attributed to librarians’ perceived lack of professional competencies to confidently deliver these wellbeing services outside the student users, or an inability to see wellbeing support for the community as within the remit of their roles. As Bladek (2021) asks, “are there professional competencies that academic librarians need to develop in order to support student wellness?” (p. 7) [63]. Similarly, Cox (2020) underscores what is also very evident from the results of this review, “academic libraries fail to convincingly define the core concept of wellbeing that the activities they are organising are trying to address” (p. 150) [27].

It is important to note that most academic libraries in this study were not health science libraries where wellbeing and health are generally the core mandate. As Cox (2020) observes in a review of academic library wellbeing initiatives for students, most academic libraries were “not merely signposting” (p. 5) [27]. Wellbeing initiatives also appeared to benefit the academic library in most cases, opening opportunities to engage with new and existing library users. Thus, it appears that academic libraries do have the capacity to present themselves as dynamic spaces where they can embody different roles, making it conducive to health promotion.

### 4.2. Strengths and Limitations

There are several strengths worth noting in this review. First, the team of researchers had quite varied backgrounds including higher education studies, health sciences, and public health. The multitude of perspectives allowed us to report more thoroughly. One limitation of this review is the difficulty in identifying clear research gaps since a majority of the citations did not provide any substantial insight into unexplored areas of the field. We did not conduct a quality appraisal of the included studies in order to be concordant with the JBI methodology. A large majority of the results relied on self-reporting, which increases the chance of bias as opposed to more reliable methods such as random physiological measurements. Wellbeing is a complex subject, and the conceptualisation of wellbeing in academic libraries as activities and initiatives, although necessary for the sake of the feasibility of the project, may have also been limiting.

### 4.3. Implications for Practice

This scoping review will allow those engaging in the delivery of activities and initiatives to be more aware of the potential of an academic library to contribute to wellbeing and perhaps design programs more intentionally, to suit not just the student cohort but the larger community as well. An important gap that remains to be addressed is the need for additional evidence to be gathered about the effectiveness and impact of wellbeing initiatives. Furthermore, it will be valuable to explore how these initiatives can bring together diverse professional groups both within the university and outside.

### 4.4. Implications for Research

One of the main gaps in our findings was the lack of systematic and peer-reviewed research into how wellbeing is conceived, defined, and evaluated in academic libraries. Future reviews may benefit from the exploration of wellbeing amongst other users such as faculty in an academic library as well. To improve the value of the research, it is important to assess the efficacy and effectiveness of the activities and initiatives in order to draw out lessons. Ongoing documentation of the wellbeing activities and initiatives might aid in showcasing the successes and challenges so that other academic libraries may learn from them.

## 5. Conclusions

This scoping review examined the existing literature on academic libraries’ user wellbeing for both student and community population groups. It revealed that academic libraries do indeed contribute to the wellbeing of their users. This review also identifies 11 different kinds of initiatives that academic libraries carry out, i.e., animal-assisted activities; facilitating dialogues about belonging and identity; fun recreational activities; study support; physical activity promotion; meditation, yoga, and mindfulness; book clubs; art exhibitions; technology and digital support; free food and tea; and health awareness. It was also found that these activities dealt with the physical, mental, and social aspects of wellbeing in various combinations. However, many of these activities target student wellbeing, and not nearly as many address community wellbeing. This scoping review encourages the reader to examine academic libraries as places conducive for wellbeing to flourish or, at minimum, as a place for health promotion to take place.

## Figures and Tables

**Table 1:** Summary of various kinds of wellbeing initiatives/activities.

Activity Types	Number of Citations	Representative Result (Please Refer to Supplementary Material S5: Characteristics of Included Studies)	Summary
(a) Meditation, yoga, and mindfulness	11	Bremer (2019) [25]; Casucci (2019) [26]; Cox, A. (2020) [27]; Flynn (2017) [28]; Funaro (2019) [29]; Karadjova-Kozhuharova (2023) [30]; Lenstra (2020) [31]; Peñaflor (2021) [32]; Porritt (2019) [33]; Rose (2015) [34]; and Walton (2018) [5]	Eleven studies reported on meditation, yoga, and mindfulness programs in their libraries. Examples include free yoga sessions for health students at University of Utah and “Breathe”—a group mindfulness program at University of Newfoundland [34]. Yet another example is dedicated meditation rooms in the library such as those at the University of Minnesota Morris [25].
(b) Animal-assisted activities	10	Cox, A (2020) [27]; Duffy (2021) [35]; Edwards (2022) [36]; Flynn (2017) [28]; Henrich (2020) [37]; Houghton (2019) [38]; Jalongo (2015) [39]; Lannon (2015) [40]; Reynolds (2011) [41]; and Walton (2018) [5]	A large majority of the reviewed studies described how animal-assisted activities were coordinated in academic libraries to help with wellbeing. Eight citations from this review were about therapy dogs and their usefulness in de-stressing during finals week or helping students relax. Participants (n = 103) in another study self-reported a decrease in feelings of tiredness after a robot petting zoo activity when the semester was in session. Another activity used owls in the library to make the space more inviting to students [38].
(c) Book clubs	7	Brewster (2023) [42], Flynn (2017) [28], Green (2022) [43], Jansen (2019) [44], Norton (2019) [45], Porritt (2019) [33], and Walton (2018) [5]	Seven citations in this review mentioned books and book clubs as a medium for addressing wellbeing amongst students. For example, the Penn State Brandywine campus library organized a book club to allow international students to share their experiences with other students on campus [44].
(d) Facilitating dialogues about identity and belonging	6	Charney (2018) [46], Everett (2018) [47], Gillum (2022) [48], Jansen (2019) [44], Porritt (2019) [33], Thomas (2019) [49]	Six citations highlighted activities within the library that facilitated dialogues on various issues including race and diversity through the Race Card project in Everrett’s (2019) [47] study or the Bring Your Own Story initiative in Gillum’s (2022) [48] study that allowed participants to discuss societal prejudices, stigmas, and stereotypes on matters such as religion and sexuality.
(e) Health awareness	6	Brewster (2023) [42], Dadzie (2016) [50], Peñaflor (2021) [32], Stone (1983) [51], Strong (2012) [52], and Thomas (2019) [49]	Health awareness activities were aimed at mostly preventing disease and death. Six citations described activities regarding the same. Mental health awareness campaigns, raising awareness about HIV through graphic novel contests, and suicide prevention campaigns are some of the activities that academic libraries have organised to raise awareness about health issues [32].
(f) Physical activity promotion	5	Clement (2018) [53], Duffy (2021) [35], Hoppenfeld (2019) [54], Lenstra (2020) [31], and Maeda (2014) [55]	Five citations reported on activities that encourage physical health by encouraging active spaces in the library. For instance, the University of Tennessee’s library installed bike and treadmill desks and balance chairs to discourage students from being sedentary in the library [53].
(g) Fun recreational activities	4	Flynn (2017) [28], Henrich (2020) [37], Newton (2011) [56], and Prichard (2020) [57]	Four citations reported on fun recreational activities such as checkers, treasure hunts, and mini golf for students to relax during the semester [56].
(h) Study support	3	Brewster (2023) [42], George (2019) [58], and Houghton (2019) [38]	Three studies highlighted activities that encourage good study habits and promote cognitive wellbeing amongst students. These included making self-help books available and organising wellbeing activities for students so they can study healthier, happier, and smarter.
(i) Art exhibitions	3	Phinney (2021) [59], Seymour (2012) [60], and Thomas (2019) [49]	Three studies described art exhibition initiatives to address wellbeing. For instance, one art exhibition in Marshall University Libraries specifically tackled the shame that is associated with mental health problems [49]. Another art exhibition responded to crimes towards the LGBTQIA community on campus [60].
(j) Free food and tea	3	Eldermire (2022) [61], Flynn (2017) [28], and Rose (2015) [34]	One initiative reported on the experience of brewing tea for students as an effort to address wellbeing [61]. Another initiative at Michigan State University provided free food to students during exam week [28].
(k) Technology and digital support	2	Cox (2013) [62] and Strong (2012) [52]	Two studies reported on activities that used technology to uplift student and community wellbeing. For example, the Senior CHAT initiative provided health awareness to the elderly through computer instruction [52].

## Data Availability

The original contributions presented in this study are included in the article/Supplementary Materials. Further inquiries can be directed to the corresponding author.

## Supplementary Materials

The following supporting information can be downloaded at https://www.mdpi.com/article/10.3390/healthcare13020179/s1: Supplementary Material S1: Search Strategy; Supplementary Material S2: Data extraction tool; Supplementary Material S3: Studies ineligible following full-text review; Supplementary Material S4: Preferred Reporting Items for Systematic reviews and Meta-Analyses extension for Scoping Reviews (PRISMA-ScR) checklist; Supplementary Material S5: Characteristics of included studies.
