# Supplementary material for: Do Academic Libraries Contribute to Students’ and Communities’ Wellbeing?: A Scoping Review
Source: Healthcare (Basel). 2025 Jan 17;13(2):179. doi: 10.3390/healthcare13020179 (PMC11765332; doi:10.3390/healthcare13020179)
Supplement: Supplementary file 1 [file healthcare-13-00179-s001.zip › healthcare-3384342-supplementary.pdf]

Supplementary Materials

Supplementary Material S1: Search Strategy

LISA
Date searched: November 29, 2023
Results generated: 2514

((abstract("academic librar*" OR "college librar*" OR "tertiary education librar*" OR "higher education librar*" OR "university librar*") OR title("academic librar*" OR "college librar*" OR "tertiary education librar*" OR "higher education librar*" OR "university librar*")) OR MAINSUBJECT.EXACT("Academic libraries")) AND ((MAINSUBJECT.EXACT("Wellness programs") OR MAINSUBJECT.EXACT("Outreach services") OR MAINSUBJECT.EXACT("Health promotion") OR MAINSUBJECT.EXACT("Public health") OR MAINSUBJECT.EXACT("Health")) OR (abstract("WellBeing" OR "well being" OR "wellbeing" OR "quality of life" OR "holistic" OR "life satisfaction*" OR "Daily Living Skill*" OR "life style*" OR "Emotional Disturbance*" OR "Wellness" OR "Safety" OR "Adjustment" OR "Morale" OR "Stress" OR "Coping" OR "Individual Power" OR "Counseling" OR "Well Being" OR "Outreach" OR "public health" OR "health") OR title("WellBeing" OR "well being" OR "wellbeing" OR "quality of life" OR "holistic" OR "life satisfaction*" OR "Daily Living Skill*" OR "life style*" OR "Emotional Disturbance*" OR "Wellness" OR "Safety" OR "Adjustment" OR "Morale" OR "Stress" OR "Coping" OR "Individual Power" OR "Counseling" OR "Well Being" OR "Outreach" OR "public health" OR "health"))) AND ((abstract("user*" OR "client*" OR "patron*" OR "student*" OR "staff" OR "facult*" OR "communit*" OR "local*" OR "library communit*" OR " member*" OR "visitor*" OR "customer*" OR "attendee*" OR "reader*" OR "researcher*" OR "borrower*" OR "scholar*") OR title("user*" OR "client*" OR "patron*" OR "student*" OR "staff" OR "facult*" OR "communit*" OR "local*" OR "library communit*" OR " member*" OR "visitor*" OR "customer*" OR "attendee*" OR "reader*" OR "researcher*" OR "borrower*" OR "scholar*")) OR (MAINSUBJECT.EXACT("Library staff") OR MAINSUBJECT.EXACT("Library users") OR MAINSUBJECT.EXACT("University graduates") OR MAINSUBJECT.EXACT("College students") OR MAINSUBJECT.EXACT("University faculty") OR MAINSUBJECT.EXACT("Community") OR MAINSUBJECT.EXACT("University professors") OR MAINSUBJECT.EXACT("Students") OR MAINSUBJECT.EXACT("University students") OR MAINSUBJECT.EXACT("School faculty")))

ERIC
Date searched: November 29, 2023
Results generated: 597

((abstract("academic librar*" OR "college librar*" OR "tertiary education librar*" OR "higher education librar*" OR "university librar*") OR title("academic librar*" OR "college librar*" OR "tertiary education librar*" OR "higher education librar*" OR "university librar*")) OR MAINSUBJECT.EXACT("Academic Libraries")) AND ((abstract("WellBeing" OR "well being" OR "wellbeing" OR "quality of life" OR "holistic" OR "life satisfaction*" OR "Daily Living Skill*" OR "life style*" OR "Emotional Disturbance*" OR "Wellness" OR "Safety" OR "Adjustment" OR "Morale" OR "Stress" OR "Coping" OR "Individual Power" OR "Counseling" OR "Well Being" OR "Outreach" OR "public health" OR "health") OR title("WellBeing" OR "well being" OR "wellbeing" OR "quality of life" OR "holistic" OR "life satisfaction*" OR "Daily Living Skill*" OR "life style*" OR "Emotional Disturbance*" OR "Wellness" OR "Safety" OR "Adjustment" OR "Morale" OR "Stress" OR "Coping" OR "Individual Power" OR "Counseling" OR "Well Being" OR "Outreach" OR "public health" OR "health")) OR (MAINSUBJECT.EXACT("Wellness") OR

MAINSUBJECT.EXACT("Literacy Education") OR MAINSUBJECT.EXACT("Outreach Programs") OR MAINSUBJECT.EXACT("Health Promotion") OR MAINSUBJECT.EXACT("Well Being") OR MAINSUBJECT.EXACT("Public Health"))) AND ((abstract("user*" OR "client*" OR "patron*" OR "student*" OR "staff" OR "facult*" OR "communit*" OR "local*" OR "library communit*" OR "member*" OR "visitor*" OR "customer*" OR "attendee*" OR "reader*" OR "researcher*" OR "borrower*" OR "scholar*") OR title("user*" OR "client*" OR "patron*" OR "student*" OR "staff" OR "facult*" OR "communit*" OR "local*" OR "library communit*" OR "member*" OR "visitor*" OR "customer*" OR "attendee*" OR "reader*" OR "researcher*" OR "borrower*" OR "scholar*")) OR (MAINSUBJECT.EXACT("Community") OR MAINSUBJECT.EXACT("Students") OR MAINSUBJECT.EXACT("Users (Information)") OR MAINSUBJECT.EXACT("College Students") OR MAINSUBJECT.EXACT("College Graduates") OR MAINSUBJECT.EXACT("Graduates") OR MAINSUBJECT.EXACT("Library Administration") OR MAINSUBJECT.EXACT("Library Role") OR MAINSUBJECT.EXACT("College Faculty") OR MAINSUBJECT.EXACT("Faculty")))

Medline
Date searched: November 29, 2023
Results generated: 239

(academic librar* or college librar* or tertiary education librar* or higher education librar* or university librar*).ti,ab.	688
(Libraries, Medical/ or Libraries/ or Library Services/ or Librarians/ or exp Libraries/) and exp Universities/	465
1 or 2	1091
("WellBeing" or "well being" or "wellbeing" or "quality of life" or "holistic" or "life satisfaction*" or "Daily Living Skill*" or "life style*" or "Emotional Disturbance*" or "Wellness" or "Safety" or "Adjustment" or "Morale" or "Stress" or "Coping" or "Individual Power" or "Counseling" or "Well Being" or "Outreach" or "public health" or "health").ti,ab.	4498852
health/ or public health/ or exp "Quality of Life"/ or Student Health Services/ or Health Promotion/	474143
4 or 5	4599342
("user*" or "client*" or "patron*" or "student*" or "staff" or "facult*" or "communit*" or "local*" or "library communit*" or "member*" or "visitor*" or "customer*" or "attendee*" or "reader*" or "researcher*" or "borrower*" or "scholar*").ti,ab.	3836989
residence characteristics/ or Students/ or faculty/	129983
7 or 8	3875076
3 and 6 and 9	239

Web of Science
Date searched: November 29, 2023
Results generated: 720

TI=("academic librar*" OR "college librar*" OR "tertiary education librar*" OR "higher education librar*" OR "university librar*") OR AB=("academic librar*" OR "college librar*" OR "tertiary education librar*" OR "higher education librar*" OR "university librar*")	17995

AND	
TI=("WellBeing" OR "well being" OR "wellbeing" OR "quality of life" OR "holistic" OR "life satisfaction*" OR "Daily Living Skill*" OR "life style*" OR "Emotional Disturbance*" OR "Wellness" OR "Safety" OR "Adjustment" OR "Morale" OR "Stress" OR "Coping" OR "Individual Power" OR "Counseling" OR "Well Being" OR "Outreach" OR "public health" OR "health") OR AB=("WellBeing" OR "well being" OR "wellbeing" OR "quality of life" OR "holistic" OR "life satisfaction*" OR "Daily Living Skill*" OR "life style*" OR "Emotional Disturbance*" OR "Wellness" OR "Safety" OR "Adjustment" OR "Morale" OR "Stress" OR "Coping" OR "Individual Power" OR "Counseling" OR "Well Being" OR "Outreach" OR "public health" OR "health")	6474011
AND	
TI=("user*" OR "client*" OR "patron*" OR "student*" OR "staff" OR "facult*" OR "communit*" OR "local*" OR "library communit*" OR " member*" OR "visitor*" OR "customer*" OR "attendee*" OR "reader*" OR "researcher*" OR "borrower*" OR "scholar*") OR AB=("user*" OR "client*" OR "patron*" OR "student*" OR "staff" OR "facult*" OR "communit*" OR "local*" OR "library communit*" OR " member*" OR "visitor*" OR "customer*" OR "attendee*" OR "reader*" OR "researcher*" OR "borrower*" OR "scholar*")	8849849
#1 AND #2 AND #3	720

Scopus
Date searched: November 29, 2023
Results generated: 1228

((ABS ("academic librar*" OR "college librar*" OR "tertiary education librar*" OR "higher education librar*" OR "university librar*") OR TITLE ("academic librar*" OR "college librar*" OR "tertiary education librar*" OR "higher education librar*" OR "university librar*"))) AND ((ABS ("WellBeing" OR "well being" OR "wellbeing" OR "quality of life" OR "holistic" OR "life satisfaction*" OR "Daily Living Skill*" OR "life style*" OR "Emotional Disturbance*" OR "Wellness" OR "Safety" OR "Adjustment" OR "Morale" OR "Stress" OR " Coping" OR "Individual Power" OR "Counseling" OR "Well Being" OR "Outreach" OR "public health" OR "health") OR TITLE ("WellBeing" OR "well being" OR "wellbeing" OR "quality of life" OR "holistic" OR "life satisfaction*" OR "Daily Living Skill*" OR "life style*" OR "Emotional Disturbance*" OR "Wellness" OR "Safety" OR "Adjustment" OR "Morale" OR "Stress" OR " Coping" OR "Individual Power" OR "Counseling" OR "Well Being" OR "Outreach" OR "public health" OR "health"))) AND ((ABS ("user*" OR "client*" OR "patron*" OR "student*" OR "staff" OR "facult*" OR "communit*" OR "local*" OR "library communit*" OR "member*" OR "visitor*" OR "customer*" OR "attendee*" OR "reader*" OR "researcher*" OR "borrower*" OR "scholar*") OR TITLE ("user*" OR "client*" OR "patron* " OR "student*" OR "staff" OR "facult*" OR "communit*" OR "local*" OR "library communit*" OR "member*" OR "visitor*" OR "customer*" OR "attendee*" OR "reader*" OR "researcher*" OR "borrower*" OR "scholar*")))

Grey Literature Search
Date searched: December 4, 2023.
Search terms for grey literature search: “academic libraries user wellbeing”.

**Name of Grey Literature source**	**Citations Generated**
International Federation of Library Associations and Institutions (IFLA)	22
Council for Australian University Libraries (CAUL)	5
American Library Association (ALA)	10
Australian Library and Information Association (ALIA)	2
Google	50
Google Scholar advanced search	50

Supplementary Material S2: Data Extraction tool

Title	First Author (Year of Publication)	Study Design	Institution	Location	Target Population	Aims/Purpose	Initiative/Activity	Key Findings	Gaps

Supplementary Material S3: Studies ineligible following full text review

Adetayo, A. J., Adekunmisi, S. R., Otonekwu, F. O., & Adesina, O. F. (2023). The role of academic libraries in facilitating friendships among students. IFLA Journal, 03400352231191540.

Reason for exclusion: No wellbeing focused activity/initiative by the library

Aghadiuno, P. C., Uzoagba, N. C., & Onyekweodiri, N. E. (2021). Awareness, attitude and impact of COVID 19 on librarians in academic libraries in Nasarawa State Nigeria. The Information Technologist, 18(1), 53. http://ezproxy.uws.edu.au/login?url=https://www.proquest.com/docview/2565199497?accountid=36155&bdid=51075&_bd=vyoXWD7tMrQ0HFnoE12Azv8QqDc%3D

Reason for exclusion: Wrong population

Akiyama, M. (2010). Community-Based Trial on Providing Cancer Information to the Public. Igaku Toshokan (Journal of the Japan Medical Library Association), 57(2), 193-198. http://ezproxy.uws.edu.au/login?url=https://www.proquest.com/docview/818633687?accountid=36155&bdid=51075&_bd=%2BcePfqBZ9JnmaE%2BGAw%2BP4Ll%2Fw5g%3D

Reason for exclusion: Not in English

Anonymous. (2020). from our READERS. American Libraries, 51(5), 8-9. http://ezproxy.uws.edu.au/login?url=https://www.proquest.com/docview/2407564632?accountid=36155&bdid=51075&_bd=YoCnLY94qdRE1JhNKYxXnZxU%2BJU%3D

Reason for exclusion: No wellbeing focused activity/initiative by the library

Arsalan, S., & Ahmed, K. M. (2021). Real-Time Covid-19 Risk Level Indicator for the Library Users.

Reason for exclusion: No wellbeing focused activity/initiative by the library

Awan, W. A. D., & Soroya, S. H. D. (2021). Foreign students' status of autonomy, environmental mastery, personal growth, positive relations, purpose in life; and association of all these with using library as a third/ community place. Library Philosophy and Practice, 0_1-34. http://ezproxy.uws.edu.au/login?url=https://www.proquest.com/docview/2557266689?accountid=36155&bdid=51075&_bd=Da31z1WcWYZhgVHfAxg2vqc2YkU%3D

Reason for exclusion: No wellbeing focused activity/initiative by the library

Ayeni, P. O., Agbaje, B. O., & Tippler, M. (2021). A Systematic Review of Library Services Provision in Response to COVID-19 Pandemic. Evidence Based Library and Information Practice, 16(3), 67. https://doi.org/https://doi.org/10.18438/eblip29902

Reason for exclusion: Wrong concept

Balzer, C. (2020). Using 3D to Make PPE. American Libraries, 51(6), 16-17. http://ezproxy.uws.edu.au/login?url=https://www.proquest.com/docview/2405660052?accountid=36155&bdid=51075&_bd=xCEojknlKWQ9gmZG0kRGCrsabUI%3D

Reason for exclusion: Wrong context

Bangani, S., & Dube, L. (2023). South African academic libraries as contributors to social justice and ubuntu through community engagement. IFLA Journal, 49(3), 541-553. https://doi.org/https://doi.org/10.1177/03400352231166751

Reason for exclusion: No wellbeing focused activity/initiative by the library

Becker, J. (2017). Active Allyship. Public Services Quarterly, 13(1), 27-31. https://doi.org/https://doi.org/10.1080/15228959.2016.1261638

Reason for exclusion: No wellbeing focused activity/initiative by the library

Bodaghi, N. B., Cheong, L. S., Zainab, A. N., & Riahikia, M. (2017). Friendly librarians: The culture of caring and inclusion experiences of visually impaired students in an academic library. Information Development, 33(3), 229-242.

Reason for exclusion: No wellbeing focused activity/initiative by the library

Bossaller, J., Oprean, D., Urban, A., & Riedel, N. (2020). A happy ambience: Incorporating ba and flow in library design. Journal of Academic Librarianship, 46(6), 1. https://doi.org/https://doi.org/10.1016/j.acalib.2020.102228

Reason for exclusion: No wellbeing focused activity/initiative by the library

Breland, M., Venturella, K., & Shapiro, S. (2023). Looking Back, Looking Ahead: Lessons Learned from the Pandemic (Are we Prepared for the Next Pandemic)? Library Leadership & Management (Online), 37(2), 1-16. http://ezproxy.uws.edu.au/login?url=https://www.proquest.com/docview/2842941335?accountid=36155&bdid=51075&_bd=Z2wApDD6oWt%2F57StwQeXPGZP1c4%3D

Reason for exclusion: No wellbeing focused activity/initiative by the library

CAUL. (2022). Making University libraries safe spaces for all students. https://www.caul.edu.au/events/making-university-libraries-safe-spaces-all-students

Reason for exclusion: No wellbeing focused activity/initiative by the library

Chunmei, Y. A. O. (2017). Discussion on the Promotion of Reading Therapy for University Libraries Based on Wechat Public Platform. Journal of Library and Information Sciences in Agriculture, 29(10), 134. https://doi.org/https://doi.org/10.13998/j.cnki.issn1002-1248.2017.10.031

Reason for exclusion: No wellbeing focused activity/initiative by the library

Chutia, R., & Sarmah, M. (2019). Psychological Well-Being Among the Gender wise Category of Users in terms of Library Anxiety at Centrally Funded Universities of Assam, India. Library Philosophy and Practice, 1-23. http://ezproxy.uws.edu.au/login?url=https://www.proquest.com/docview/2236689059?accountid=36155&bdid=51075&_bd=DLUMe9n76F42seNYirZGKMK490E%3D

Reason for exclusion: No wellbeing focused activity/initiative by the library

Corrall, S. (2022). The Social Mission of Academic Libraries in Higher Education.

Reason for exclusion: No wellbeing focused activity/initiative by the library

Cox, A., & Brewster, L. (2022). Aligned but not integrated: UK academic library support to mental health and well-being during COVID-19. Library Management, 43(1/2), 108-127. https://doi.org/https://doi.org/10.1108/LM-09-2021-0075

Reason for exclusion: No wellbeing focused activity/initiative by the library

Cox, A. M., & Brewster, L. (2021). Services for Student Well-Being in Academic Libraries: Three Challenges. New Review of Academic Librarianship, 27(2), 149-164. https://doi.org/https://doi.org/10.1080/13614533.2019.1678493

Reason for exclusion: No wellbeing focused activity/initiative by the library

Danarsiwi Tri, L., & Nurfarawahidah, B. Library for the homeless: A case study of a Shelter House and a School for Homeless in Indonesia and Malaysia.

Reason for exclusion: Wrong Context

Decker, E. N. (2021). Reaching Academic Library Users during the COVID-19 Pandemic: New and Adapted Approaches in Access Services. Journal of Access Services, 18(2), 77-90. https://doi.org/https://doi.org/10.1080/15367967.2021.1900740

Reason for exclusion: No wellbeing focused activity/initiative by the library

Desrosiers, C. (2019). searchBOX: simplifying accessible content search. ALISS Quarterly, 14(2), 18. http://ezproxy.uws.edu.au/login?url=https://www.proquest.com/docview/2182478723?accountid=36155&bdid=51075&_bd=%2BWyW4NNVmk%2BZpb3nFFACLm70Qew%3D

Reason for exclusion: No wellbeing focused activity/initiative by the library

Dewan, P. (2023). Leisure Reading as a Mindfulness Activity: The Implications for Academic Reference Librarians. The Reference Librarian, 64(1), 1-16. https://doi.org/https://doi.org/10.1080/02763877.2022.2156968

Reason for exclusion: No wellbeing focused activity/initiative by the library

Dfree. (2020). New from ACRL - "Student Wellness and Academic Libraries: Case Studies and Activities for Promoting Health and Success". News and Press Center. https://www.ala.org/news/member-news/2020/11/new-acrl-student-wellness-and-academic-libraries-case-studies-and-activities

Reason for exclusion: No wellbeing focused activity/initiative by the library

Dfree. (2021). Keeping Up With . . . Trauma-Informed Pedagogy. Association of College & Research Libraries (ACRL). https://www.ala.org/acrl/publications/keeping_up_with/trauma-informed-pedagogy

Reason for exclusion: Wrong context

Dudgeon, P., Milroy, H., & Walker, R. Working Together: Aboriginal and Torres Strait Islander Mental Health and Wellbeing.

Reason for exclusion: Wrong context

Duesing, A. (2009). Academic Health Sciences Library Outreach and a State Cancer Coalition: Collaborative Efforts in the Cancer Battle. Journal of consumer health on the Internet, 13(3), 237. https://doi.org/https://doi.org/10.1080/15398280903119879

Reason for exclusion: No wellbeing focused activity/initiative by the library

Dunkel, C. (2012). What Does Grandpa Want from Us? Senior Citizens as Users of Academic Libraries. BuB Forum Bibliothek und Information, 64(11-12), 771-773. http://ezproxy.uws.edu.au/login?url=https://www.proquest.com/docview/1438546952?accountid=36155&bdid=51075&_bd=2sZs0JC7%2BdsHVTrDDPI2wQj3ys4%3D

Reason for exclusion: No wellbeing focused activity/initiative by the library

Edwards, M. M., & Thornton, E. (2013). Library Outreach: Introducing Campus Childcare Providers to the Academic Library. Education Libraries, 36(2), 4-16. http://ezproxy.uws.edu.au/login?url=https://www.proquest.com/scholarly-journals/library-outreach-introducing-campus-childcare/docview/1697505471/se-2?accountid=36155

Reason for exclusion: Wrong Population

Fagiolo, S. (2021). Student Wellness and Academic Libraries: Case Studies and Activities for Promoting Health and Success, edited by Sara Holder and Amber Lannon. Canadian Journal of Academic Librarianship, 7, n/a. https://doi.org/https://doi.org/10.33137/cjal-rcbu.v7.37240

Reason for exclusion: Wrong context

Falloon, K. (2015). A case study in practice: Providing accessibility for persons with disabilities at the College of Staten Island Library. Advances in Librarianship, 40, 89-107. https://doi.org/10.1108/S0065-283020150000040014

Reason for exclusion: Wrong concept

Flack, M. (2021). Sustainable Development Goals: Stretch Targets for Australian Libraries 2020-2030. ALIA Library. https://read.alia.org.au/sustainable-development-goals-stretch-targets-australian-libraries-2020-2030

Reason for exclusion: Wrong concept

Fraser, K. L., & Bartlett, J. C. P. (2018). Fear at First Sight: Library Anxiety, Race, and Nova Scotia. Partnership: the Canadian Journal of Library and Information Practice and Research, 13(2), 1-22. https://doi.org/https://doi.org/10.21083/partnership.v13i2.4366

Reason for exclusion: No wellbeing focused activity/initiative by the library

Free, D. (2022). In the News. College & Research Libraries News, 83(1), 3. https://doi.org/https://doi.org/10.5860/crln.83.1.2

Reason for exclusion: No wellbeing focused activity/initiative by the library

Libraries as Wellbeing Supportive Spaces in Contemporary Schools. https://www.tandfonline.com/doi/full/10.1080/01930826.2021.1947056

Reason for exclusion: Wrong population

Geng, P., & He, W. (2020). Sustained service in academic library during the outbreak of emergency epidemic diseases. Zhonghua Yi Xue Tu Shu Qing Bao Za Zhi = Chinese Journal of Medical Library and Information Science, 29(5), 71.

http://ezproxy.uws.edu.au/login?url=https://www.proquest.com/docview/2457700828?accountid=36155&bdid=51075&_bd=bqaRBhZDYH5iAH%2F%2BP1wXrnNmytU%3D

Reason for exclusion: Not in english

Goodingcall, A. (2022). Mental Health in the Library. American Libraries, 53(1/2), 52-53. http://ezproxy.uws.edu.au/login?url=https://www.proquest.com/docview/2620024367?accountid=36155&bdid=51075&_bd=QqddaKfAH1P1xhpGif0xFdTDsyc%3D

Reason for exclusion: Wrong population

Gorantonaki, E. J., & Uzzell, D. (2018). Searching for coziness in a university library: When psychology and design come together. Journal of Architectural and Planning Research, 35(2), 91-105. https://www.scopus.com/inward/record.uri?eid=2-s2.0-85069645051&partnerID=40&md5=571bd47188ff256628870ea1c720d611

Reason for exclusion: No wellbeing focused activity/initiative by the library

Green, M. P. (2020). Inclusive Library Service to Individuals with Mental Illnesses and Disorders. International Journal of Information, Diversity and Inclusion, 4, 119-126. https://doi.org/10.33137/ijidi.v4i1.32500

Reason for exclusion: No wellbeing focused activity/initiative by the library

Hall, K., & McAlister, S. (2021). Library Services and Resources in Support of Mental Health: A Survey of Initiatives in Public and Academic Libraries. Journal of Library Administration, 61(8), 936-946. https://doi.org/https://doi.org/10.1080/01930826.2021.1984137

Reason for exclusion: Wrong context

Health and wellbeing. (2014). St Antony's College. https://www.sant.ox.ac.uk/current-members/health-and-wellbeing

CAUL. (2022). Making University libraries safe spaces for all students. https://www.caul.edu.au/events/making-university-libraries-safe-spaces-all-students

Reason for exclusion: Wrong context

Hefner, J. A., & Rhodes, L. G. (1987). Excellence in Education: Libraries Facilitating Learning for Minority Students. http://ezproxy.uws.edu.au/login?url=https://www.proquest.com/reports/excellence-education-libraries-facilitating/docview/63263637/se-2?accountid=36155

Reason for exclusion: Wrong concept

Hlavcheva, Y. M., Odnovolykova, O. V., & Novosolova, S. Y. (2022). Safety as One of the Most Important Components of the Digital Competence System in Wartime Conditions.

Reason for exclusion: Wrong concept

Hodge, M. (2022). Library Mood: Re-Creating the Library Experience from Home. portal: Libraries and the Academy, 22(1), 227-240. https://doi.org/https://doi.org/10.1353/pla.2022.0002

Reason for exclusion: No wellbeing focused activity/initiative by the library

Holmes, S., & Yue, J. (2000). Senior citizens and the academic library: building a stronger connection. Colorado Libraries, 26(3), 24-26. http://ezproxy.uws.edu.au/login?url=https://www.proquest.com/docview/57533344?accountid=36155&bdid=51075&_bd=zzruvOTGaroOHIwIdX7iJBxQSMc%3D

Reason for exclusion: No wellbeing focused activity/initiative by the library

Hunter, C. (2021). How can academic libraries help support students' mental health? Reflective Professional, 1.

Reason for exclusion: No wellbeing focused activity/initiative by the library

Husaini, H., Shuhidan, S. M., Daud, S. C., & Priyanto, I. (2022). Library Therapeutic Landscape Support for Covid-19 Pandemic-Related Student Well-Being in Academic Libraries

Reason for exclusion: No wellbeing focused activity/initiative by the library

Jameson, J., & Duhon, L. (2023). A 10-year follow-up survey of US academic libraries highlights the COVID-19 experience and greater interest in health information outreach. Health information and libraries journal, 40(3), 275-291. https://doi.org/https://doi.org/10.1111/hir.12446

Reason for exclusion: Wrong context

Johnstone, J. (2005). Employment of Disabled Persons in the Academic Library Environment. Australian Library Journal, 54(2), 156-163. http://ezproxy.uws.edu.au/login?url=https://www.proquest.com/scholarly-journals/employment-disabled-persons-academic-library/docview/1322250866/se-2?accountid=36155

Reason for exclusion: Wrong concept

Jorge, I. M. P., Klein, A. A., Avila, A. D., & Sakowicz, E. G. (2016). The occupational therapist as a consultant on a university library layout adequacy. CADERNOS DE TERAPIA OCUPACIONAL DA UFSCAR, 24(3), 509-518. https://doi.org/10.4322/0104-4931.ctoAO0711

Reason for exclusion: No wellbeing focused activity/initiative by the library

Lackey, M. M. S. I., Swogger, S. M., & McGraw, K. A. M. A. M. L. S. (2014). Building capacity in a health sciences library to support global health projects*. Journal of the Medical Library Association, 102(2), 92. https://doi.org/https://doi.org/10.3163/1536-5050.102.2.006

Reason for exclusion: Wrong context

Lee, Y. S. (2014). Collaborative activities and library indoor environmental quality affecting performance, health, and well-being of different library user groups in higher education. Facilities, 32(3), 88-103. https://doi.org/10.1108/F-02-2013-0012

Reason for exclusion: Wrong context

Lenn, K. (1996). Library Services to Disabled Students: Outreach and Education. http://ezproxy.uws.edu.au/login?url=https://www.proquest.com/reports/library-services-disabled-students-outreach/docview/62687588/se-2?accountid=36155

Reason for exclusion: No wellbeing focused activity/initiative by the library

Liu, D., Zhang, X.-g., & Ren, S.-m. (2018). User profile-based bibliotherapy model in academic library. Zhonghua Yi Xue Tu Shu Qing Bao Za Zhi = Chinese Journal of Medical Library and Information Science, 27(7). http://ezproxy.uws.edu.au/login?url=https://www.proquest.com/docview/2176698170?accountid=36155&bdid=51075&_bd=iENdAt4CTNEPeVc30l5iq0OlQrY%3D

Reason for exclusion: Not in english

Livingston, C. B., & Grombly, A. (2020). Thinking About the Unthinkable: A Survey of Active Shooter Preparedness in Library Environments. Library Leadership & Management (Online), 35(1), 1-17. http://ezproxy.uws.edu.au/login?url=https://www.proquest.com/docview/2491232281?accountid=36155&bdid=51075&_bd=0YkL%2Bm1Z4q5wwhvXlR2Vbn%2BnAks%3D

Reason for exclusion: Wrong population

Lowry, C. B. (2003). Chapter 5. Creating a culture of security in the university of maryland libraries. Journal of Library Administration, 38(1-2), 49-57. https://doi.org/10.1300/J111v38n01_06

Reason for exclusion: Wrong concept

MacAdam, B., & Nichols, D. P. (1989). Peer Information Counseling: An Academic Library Program for Minority Students. Journal of Academic Librarianship, 15(4), 204-209. http://ezproxy.uws.edu.au/login?url=https://www.proquest.com/scholarly-journals/peer-information-counseling-academic-library/docview/63043217/se-2?accountid=36155

Reason for exclusion: No wellbeing focused activity/initiative by the library

McMullen, K. D., & Kane, L. T. (2008). Better Safe than Sorry: Panic Buttons as a Security Measure in an Academic Medical Library. Public Services Quarterly, 4(4), 391-396. http://ezproxy.uws.edu.au/login?url=https://www.proquest.com/scholarly-journals/better-safe-than-sorry-panic-buttons-as-security/docview/742862402/se-2?accountid=36155

Reason for exclusion: Wrong concept

Mills, C. P., Paladino, E. B., & Klentzin, J. C. (2015). Student veterans and the academic library. Reference Services Review, 43(2), 262. https://doi.org/https://doi.org/10.1108/RSR-10-2014-0049

Reason for exclusion: No wellbeing focused activity/initiative by the library

Miranda, C., Horton, W., & Miranda, C. (1983). The health sciences library in the community college: supporting the Associate Degree Nursing Program. Community and Junior College Libraries, 2(2). http://ezproxy.uws.edu.au/login?url=https://www.proquest.com/docview/57254941?accountid=36155&bdid=51075&_bd=Db%2FIAOfhDu03Ern5EDKtCSucXlQ%3D

Reason for exclusion: Wrong concept

Miščin, Ž., & Gabriel, D. M. (2017). National campaign for persons with reading difficulties and dyslexia „I want to read too!“. Vjesnik Bibliotekara Hrvatske, 60(1), 289-309. https://www.scopus.com/inward/record.uri?eid=2-s2.0-85035129855&partnerID=40&md5=e799a72b3092a8aa85de3210e209f11f

Reason for exclusion: Wrong context

Monica Mensah, D., Dadzie, P. S., & Gyesi, K. (2022). Academic Libraries in Ghana and Their Strategies for Coping with the First Wave of the COVID-19 Pandemic. International Journal of Librarianship, 7(2), 30. https://doi.org/https://doi.org/10.23974/ijol.2022.vol7.2.232

Reason for exclusion: No wellbeing focused activity/initiative by the library

Nasiruddin, M. (2013). Shelter-Based Community Libraries: In Search of Alternative Livelihoods for Pavement Dwellers in Dhaka City. US-China Education Review B, 3(1), 62-70. http://ezproxy.uws.edu.au/login?url=https://www.proquest.com/scholarly-journals/shelter-based-community-libraries-search/docview/1322241484/se-2?accountid=36155

Reason for exclusion: Wrong context

Nieradko, B., & Borzecki, A. (2003). Exercise behavior, sleep habits and time management among students of the Medical University of Lublin. Annales Universitatis Mariae Curie-Sklodowska. Sectio D: Medicina, 58(1), 358-361.

Reason for exclusion: Wrong context

Nixon, M. L. (2015). Safety doesn't happen by accident: Disaster planning at the university of Pittsburgh. In (pp. 184-206). https://doi.org/10.4018/978-1-4666-8624-3.ch009

Reason for exclusion: No wellbeing focused activity/initiative by the library

Norlin, E. (2001). University goes back to basics to reach minority students. American Libraries, 32(7), 60-62. http://ezproxy.uws.edu.au/login?url=https://www.proquest.com/docview/57497726?accountid=36155&bdid=51075&_bd=5wMh4iShD9YBdJA2YZHo8BzQcZU%3D

Reason for exclusion: No wellbeing focused activity/initiative by the library

Otto, J. L., Meade, Q. H., Stafford, J. L., & Wahler, P. (2016). Library Lights Out: A creative collaboration between the library, students, and university housing. Digital Library Perspectives, 32(3), 192-208. https://doi.org/10.1108/DLP-09-2015-0018

Reason for exclusion: No wellbeing focused activity/initiative by the library

Peng, T., Fang, Z., Zheng, Z., Ji, Z., & Li, Q. (2020). Research on Thermal Comfort of University Libraries in Summer of Guangzhou.

Reason for exclusion: No wellbeing focused activity/initiative by the library

Poljak, L., Webster, B. M., & Kiner, R. (2023). Exploring belonging through photovoice: examining the impact of space design on diverse student populations in an academic library. Performance Measurement and Metrics, 24(3/4), 195-210. https://doi.org/https://doi.org/10.1108/PMM-08-2023-0023

Reason for exclusion: No wellbeing focused activity/initiative by the library

Prokopenko, L., & Skachenko, O. (2023). Meeting Users’ Information Needs Online during the Russian-Ukrainian War. Portal : Libraries and the Academy, 23(4), 655. https://doi.org/https://doi.org/10.1353/pla.2023.a908696

Reason for exclusion: Wrong context

Ragsdale, K. W., & Simpson, J. (1996). Being on the safe side. College and Research Libraries News, 57(6), 351-354. http://ezproxy.uws.edu.au/login?url=https://www.proquest.com/docview/57406413?accountid=36155&bdid=51075&_bd=32CQLx2NjQvcahuzHbXyku%2B%2BXOo%3D

Reason for exclusion: Wrong concept

Ramsey, E., & Aagard, M. C. (2018). Academic libraries as active contributors to student wellness. College & Undergraduate Libraries, 25(4), 328. https://doi.org/https://doi.org/10.1080/10691316.2018.1517433

Reason for exclusion: No wellbeing focused activity/initiative by the library

Rchristopher. (2018). How libraries can support community health and wellbeing through bibliotherapy. News and Press Center. https://www.ala.org/news/member-news/2018/11/how-libraries-can-support-community-health-and-wellbeing-through-bibliotherapy

Reason for exclusion: Wrong context

Richardson, L. M., Renner, B. R., Ottosen, T., & Goldstein, A. O. (2019). A Library and a Radio Show: The Story of a Successful Partnership at 10 Years and Counting. Journal of Library Administration, 59(4), 395. https://doi.org/https://doi.org/10.1080/01930826.2019.1593713

Reason for exclusion: Wrong context

Rosenzweig, M., & Schnitzer, A. E. (2009). Partners for Excellence: How the University of Michigan Health Sciences Libraries Assisted the Ann Arbor Public Schools in Their Health and Wellness Curriculum. Journal of consumer health on the Internet, 13(4), 359. https://doi.org/https://doi.org/10.1080/15398280903341002

Reason for exclusion: No wellbeing focused activity/initiative by the library

Rutledge, L., & LeMire, S. (2016). Beyond Disciplines: Providing Outreach to Underserved Groups by Demographic. Public Services Quarterly, 12(2), 113-124. https://doi.org/https://doi.org/10.1080/15228959.2016.1157565

Reason for exclusion: No wellbeing focused activity/initiative by the library

Samantha Schmehl, H. Connecting Individuals With Social Services: The Library’s Role. Reference as service and place.

Reason for exclusion: Wrong context

Samson, S. (2017). Student veterans in the academic library. College & Undergraduate Libraries, 24(1), 80. https://doi.org/https://doi.org/10.1080/10691316.2015.1125318

Reason for exclusion: No wellbeing focused activity/initiative by the library

Sharing good practice on supporting wellbeing: NHS libraries and Academic Libraries North - LKS North. https://www.lksnorth.nhs.uk/events/sharing-good-practice-on-supporting-wellbeing-nhs-libraries-and-academic-libraries-north/

Reason for exclusion: No wellbeing focused activity/initiative by the library

Sheih, S.-M. C., & Hung, H.-J. (2013). A Study on Undergraduate Demand for Developmental Bibliotherapeutic Service in University Libraries. Journal of Library and Information Science

Research, 8(1), 29-68. http://ezproxy.uws.edu.au/login?url=https://www.proquest.com/docview/1629324614?accountid=36155&bdid=51075&_bd=e6VqTUV7o1seGHsa031SSwi0mn0%3D

Reason for exclusion: No wellbeing focused activity/initiative by the library

Shotick, K. (2021). Student wellness and academic libraries: case studies and activities for promoting health and success. Public Services Quarterly, 17(3), 185-186. https://doi.org/https://doi.org/10.1080/15228959.2021.1933785

Reason for exclusion: Wrong concept

Shumaker, D. (2021). Beyond Coping: Libraries Stepping Up to Meet Community Needs During the Pandemic. Information Today, 38(2), 12-14. http://ezproxy.uws.edu.au/login?url=https://www.proquest.com/docview/2572294272?accountid=36155&bdid=51075&_bd=ohDiItIhlUAwAfIKPgoFrV93iqw%3D

Reason for exclusion: Wrong context

Sidorko, P. E., & Yang, T. T. (2011). Knowledge exchange and community engagement: an academic library perspective. Library Management, 32(6/7), 385. https://doi.org/https://doi.org/10.1108/01435121111158538

Reason for exclusion: No wellbeing focused activity/initiative by the library

Somaratna, S. D. (2021). Student Counsellors' Perception on Initiating Bibliotherapy Service in the University Environment. Journal of the University Librarians Association of Sri Lanka, 24(2), 27. https://doi.org/https://doi.org/10.4038/jula.v24i2.8046

Reason for exclusion: Wrong context

Sylka, C., Gilpin, C., & Fama, K. (2020). Peer Assisted Study Sessions: How Academic Libraries Can Influence Student Well-Being through Academic Programs and Intentional Partnerships.

Reason for exclusion: No wellbeing focused activity/initiative by the library

Tella, A., Akande, F. T., Adigun, G. O., Odunola, O., & Stella, N. (2017). Library and information services targeting poverty alleviation for sustainable human development. Mousaion, 35(1), 28. http://ezproxy.uws.edu.au/login?url=https://www.proquest.com/docview/2052878408?accountid=36155&bdid=51075&_bd=yXGxh8%2FfVROsKR7RFjxBOuUrDQA%3D

Reason for exclusion: No wellbeing focused activity/initiative by the library

Thomas, S., & Lovelace, K. (2019). Ending the Silence: Utilizing Personal Experiences to Enhance a Library Mental Health Initiative. Journal of Intellectual Freedom and Privacy, 4(2), 3. https://doi.org/https://doi.org/10.5860/jifp.v4i2.7079

Reason for exclusion: No wellbeing focused activity/initiative by the library

Wan, Y. P., & Zhai, Y. F. (2022). Investigation and analysis on the demand of college students' readers for library mental health resources - a case study of Hainan university library. Psychiatria Danubina, 34, s673-s674.

Reason for exclusion: No wellbeing focused activity/initiative by the library

Wellbeing resources | Senate House Library | University of London. https://www.london.ac.uk/about/services/senate-house-library/using-library/services-help/wellbeing-resources

Reason for exclusion: No wellbeing focused activity/initiative by the library

Wilmoth, W. S. (2008). Serving Sexual Assault Survivors in the Academic Library: Using the Tools of Crisis Intervention and Empowerment Counseling in the Reference Interview. Georgia Library Quarterly, 45(1), 9-13. http://ezproxy.uws.edu.au/login?url=https://www.proquest.com/docview/57496702?accountid=36155&bdid=51075&_bd=2OHWOwSaC5Z8GInNMKpI26LW4ZA%3D

Reason for exclusion: No wellbeing focused activity/initiative by the library

Wilson, A. J., Staley, C., Davis, B., & Blair, A. (2023). Libraries advancing health equity:a literature review. Reference Services Review, 51(1), 65-76. https://doi.org/https://doi.org/10.1108/RSR-09-2022-0037

Reason for exclusion: No wellbeing focused activity/initiative by the library

Wise, M. J. (2018). Naps and Sleep Deprivation: Why Academic Libraries Should Consider Adding Nap Stations to Their Services for Students. New Review of Academic Librarianship, 24(2), 192-210. https://doi.org/https://doi.org/10.1080/13614533.2018.1431948

Reason for exclusion: No wellbeing focused activity/initiative by the library

Womack, H. D. (2019). Successful campus outreach for academic libraries: building community through collaboration. Technical Services Quarterly, 36(4), 429. https://doi.org/https://doi.org/10.1080/07317131.2019.1664123

Reason for exclusion: No wellbeing focused activity/initiative by the library

Wylie, N. (2021). How Are You? How Are You Really? A Focus On Staff & Student Wellbeing In The Context Of A Global Pandemic And Beyond. ALISS Quarterly, 17(1), 13. http://ezproxy.uws.edu.au/login?url=https://www.proquest.com/docview/2599115886?accountid=36155&bdid=51075&_bd=odWJBRlbJHC%2B27OTWch486YykLU%3D

Reason for exclusion: Wrong population

Xie, I., Babu, R., Lee, T. H., Wang, S., & Lee, H. S. (2021). Coping tactics of blind and visually impaired users: Responding to help-seeking situations in the digital library environment. Information Processing & Management, 58(5), 1. https://doi.org/https://doi.org/10.1016/j.ipm.2021.102612

Reason for exclusion: Wrong context

Zhang, Y., Wu, Y. Q., & Liu, L. (2013). Human library and Psychological Health of College Students

Reason for exclusion: Record unavailable

Supplementary Material S4:
Preferred Reporting Items for Systematic reviews and Meta-Analyses extension for Scoping Reviews (PRISMA-ScR) Checklist

SECTION	ITEM	PRISMA-ScR CHECKLIST ITEM	REPORTED ON PAGE #
**TITLE**			
Title	1	Identify the report as a scoping review.	1
**ABSTRACT**			
Structured summary	2	Provide a structured summary that includes (as applicable): background, objectives, eligibility criteria, sources of evidence, charting methods, results, and conclusions that relate to the review questions and objectives.	2
**INTRODUCTION**			
Rationale	3	Describe the rationale for the review in the context of what is already known. Explain why the review questions/objectives lend themselves to a scoping review approach.	3-5
Objectives	4	Provide an explicit statement of the questions and objectives being addressed with reference to their key elements (e.g., population or participants, concepts, and context) or other relevant key elements used to conceptualize the review questions and/or objectives.	5
**METHODS**			
Protocol and registration	5	Indicate whether a review protocol exists; state if and where it can be accessed (e.g., a Web address); and if available, provide registration information, including the registration number.	6
Eligibility criteria	6	Specify characteristics of the sources of evidence used as eligibility criteria (e.g., years considered, language, and publication status), and provide a rationale.	7
Information sources*	7	Describe all information sources in the search (e.g., databases with dates of coverage and contact with authors to identify additional sources), as well as the date the most recent search was executed.	6
Search	8	Present the full electronic search strategy for at least 1 database, including any limits used, such that it could be repeated.	29-32
Selection of sources of evidence†	9	State the process for selecting sources of evidence (i.e., screening and eligibility) included in the scoping review.	7
Data charting process‡	10	Describe the methods of charting data from the included sources of evidence (e.g., calibrated forms or forms that have been tested by the team before their use, and whether data charting was done independently or in duplicate) and any processes for obtaining and confirming data from investigators.	8
Data items	11	List and define all variables for which data were sought and any assumptions and simplifications made.	11
Critical appraisal of individual sources of evidence§	12	If done, provide a rationale for conducting a critical appraisal of included sources of evidence; describe the methods used and how this information was used in any data synthesis (if appropriate).	N/A

SECTION	ITEM	PRISMA-ScR CHECKLIST ITEM	REPORTED ON PAGE #
Synthesis of results	13	Describe the methods of handling and summarizing the data that were charted.	8
**RESULTS**			
Selection of sources of evidence	14	Give numbers of sources of evidence screened, assessed for eligibility, and included in the review, with reasons for exclusions at each stage, ideally using a flow diagram.	10
Characteristics of sources of evidence	15	For each source of evidence, present characteristics for which data were charted and provide the citations.	11
Critical appraisal within sources of evidence	16	If done, present data on critical appraisal of included sources of evidence (see item 12).	N/A
Results of individual sources of evidence	17	For each included source of evidence, present the relevant data that were charted that relate to the review questions and objectives.	12
Synthesis of results	18	Summarize and/or present the charting results as they relate to the review questions and objectives.	12
**DISCUSSION**			
Summary of evidence	19	Summarize the main results (including an overview of concepts, themes, and types of evidence available), link to the review questions and objectives, and consider the relevance to key groups.	19
Limitations	20	Discuss the limitations of the scoping review process.	20
Conclusions	21	Provide a general interpretation of the results with respect to the review questions and objectives, as well as potential implications and/or next steps.	21
**FUNDING**			
Funding	22	Describe sources of funding for the included sources of evidence, as well as sources of funding for the scoping review. Describe the role of the funders of the scoping review.	N/A

JBI = Joanna Briggs Institute; PRISMA-ScR = Preferred Reporting Items for Systematic reviews and Meta-Analyses extension for Scoping Reviews.
* Where *sources of evidence* (see second footnote) are compiled from, such as bibliographic databases, social media platforms, and Web sites.
† A more inclusive/heterogeneous term used to account for the different types of evidence or data sources (e.g., quantitative and/or qualitative research, expert opinion, and policy documents) that may be eligible in a scoping review as opposed to only studies. This is not to be confused with *information sources* (see first footnote).
‡ The frameworks by Arksey and O'Malley (6) and Levac and colleagues (7) and the JBI guidance (4, 5) refer to the process of data extraction in a scoping review as data charting.
§ The process of systematically examining research evidence to assess its validity, results, and relevance before using it to inform a decision. This term is used for items 12 and 19 instead of "risk of bias" (which is more applicable to systematic reviews of interventions) to include and acknowledge the various sources of evidence that may be used in a scoping review (e.g., quantitative and/or qualitative research, expert opinion, and policy document).

From: Tricco AC, Lillie E, Zarin W, O'Brien KK, Colquhoun H, Levac D, et al. PRISMA Extension for Scoping Reviews (PRISMAScR): Checklist and Explanation. Ann Intern Med. 2018;169:467–473. doi: 10.7326/M18-0850.

Supplementary Material S5: Characteristics of included studies.

Result Number	Title	First Author (Year of Publication)	Study Design	Institution	Location	Target Population		Aim/Purpose	Initiative/ Activity	Key Findings	Gaps
1	Student well-being matters: Academic library support for the whole student	Bladek, M. (2021) [63]	Comprehensive review	John Jay College of Criminal Justice	New York, USA	General student population		The purpose of the study was to showcase how academic libraries are using their library collections, spaces and service to promote wellbeing, offer critiques on this trend and make recommendations for implementing effective wellbeing initiatives.	A range of wellbeing initiatives were described in the literature review. These included finals week activities and initiative that ran in libraries during COVID	Students' overall wellbeing suffered during the COVID-19 pandemic, particularly in managing stress and anxiety. The authors found that the wellbeing initiatives promoted wellbeing amongst students by being responsive to students' needs.	
2	Reflecting on student mental health: Creating a meditation room in a small college library	Bremer, P. (2019) [25]	Case study, Essay	University of Minnesota-Morris	USA	General student population		Activity was initiated to help with student depression and mental health issues	Briss Library meditation room	The library recognised the advantage of having a more permanent space for reflection and relaxation. Students took advantage of the wellbeing resources in the meditation room and feedback from students were positive. Usage data indicated that the meditation room was used 13% of all the time it could be possibly checked in.	Developing partnerships to take over the running of the initiative was found to be a challenge.

3	Taking a 'Whole-University' Approach to Student Mental Health: The Contribution of Academic Libraries	Brewster, L. (2023) [42]	Survey	Sector Wide	UK	General student population		The aim of the study was to see how #Stepchange policy manifest in the academic library.	Some of the wellbeing activities described in the paper that were in practice were: mental health promotional campaigns, providing self-help books, encouraging good study habits, signposting	The translation between high level policy and grassroots level initiatives are not always straightforward. There also appears to be a mismatch between policy and what is actioned in practice. The study showed that while #Stepchange aimed to promote a whole-university approach to students ' wellbeing, library only intervened where they had relevant expertise.	Further research could be done to investigate how the whole university approach may be approached through bringing together of different professional groups.
4	A health sciences library promotes wellness with free yoga	Casucci, T (2019) [26]	Case Study	Spencer S. Eccles Health Sciences Library, University of Utah	Utah, USA	Students (cohort of health sciences and medicine students mostly)		To improve wellbeing amongst the health science student cohort through a 10-week free yoga session trial.	Free Yoga	Yoga provides students a welcome midday break and the event was feasibly and easy to manage means to improving student mental health.<br><br>The initiative was found to brings new users in the library and increase awareness about library services.<br><br>Impact:<br>The program recorded thirty-nine unique attendees to the yoga program.<br><br>There were several reflections from participants. Participant wrote:<br>"It is the perfect opportunity to break away from work". "Yoga is something that is so good for the mind and body and I think it is lovely that we get to participate in it here."<br>"It…rejuvenates me" P.82.<br>Others wrote: "I loved the calm	The paper identified a major need to promote mental health awareness and stress reduction. A challenge the authors identified was that oftentimes it's difficult to locate a physical space in academic libraries to run yoga or wellness initiatives.

										atmosphere…it was easy to focus and relax here.”	
5	Contemplative Pedagogy: Building resilience in academic libraries	Charney, M. (2018) [46]	Conceptual Paper	Sector Wide	USA	General student population	Community	Mindfulness-based practices and pedagogies builds resilience for students	Truth: Finding your Voice around the climate crise was an initiative described in the paper.	Conversations in the event ignited interesting discussions and it gave students the space to be with peers who felt similarly about the effect of climate change. Contemplative pedagogy will position libraries to contribute to building resilience even more.60 individuals attended the first event of Talking Truth.	
6	Reading, writing, and…running? Assessing active space in libraries	Clement, K. A. (2018) [53]	Ethnographic observational study and survey	University of Tennessee	Tennessee, USA	General student population		This paper assessed how active spaces were set up and used within the library amongst its student users.	Active spaces within the academic library that are stocked with equipment such as standing desks, treadmill and cycling desks and balance chairs to encourage	The study finds that users find active learning spaces beneficial to their physical and mental wellbeing.	More research needs to be done to have a better idea of usage patterns of active spaces in the library.

									physical activity.		
7	Engaging the Homeless through Technology and Information Literacy	Cox F.M. (2013) [62]	Case Study	University of Louisville	West Louisville, USA		Community	To improve quality of life for West Louisville residents in four areas i.e., education, health, economic development and social and human services- (p74-75)	Wayside 100 computing skills class that focused on use of Word, opening an email, creating a resume, applying for Job online and evaluating information (p 77)	24 students attended the first Wayside 100 computer class.<br><br>“Partnership between the library and community was unique because it places the universities' libraries in the position to work with some of the most vulnerable and unskilled student in Louisville and to aid them in their pursuit of education and lifelong learning.” (p81).<br><br>It facilitated learning of information literacy skills, opened opportunities for skills development and networking, and supported university recruitment.	
8	Library support for student mental health and well-being in the UK: Before and during the COVID-19 pandemic	Cox, A. (2020) [27]	Survey	Sector-Wide within UK	UK	General student population		This aim of the study was to examine how academic libraries in the United Kingdom have supported student wellbeing pre-COVID and post-COVID, and how the services were conceptualised and measured for success.	The authors created a 20-question survey to gather data on wellness initiatives in academic libraries. Activities that the library was conducting before Covid 19 were along the themes of mindfulness, animal-petting, yoga, and craft classes.	Findings highlight that academic libraries were actively developing wellbeing-related activities and “not merely falling into the line of signposting although that was happening too”.<br><br>The responses from the survey suggested that any anxiety post-Covid was mostly related to the shift to digital content for study and worries related to accessing resources rather that the pandemic itself.	There appeared to be a gap in the available evidence about the efficacy and popularity of wellbeing initiatives and how wellbeing is conceived in them.

9	Ensuring healthy lives and promoting well-being for all: the role of Ghanaian academic libraries in achieving the 2030 Agenda for Sustainable development	Dadzie, P.S. (2016) [50]	Survey	Sector Wide	Ghana		Community	The aim of this study was to assess awareness about the SDGs, particularly SDG3 amongst university librarians and to examine the role and process of academic libraries to achieve SDG3.	A range of wellbeing initiatives were mentioned including health science libraries. However, these were activities that discharged as part of the role of being a health science library attached to a medical or health science school.	Academic Libraries that serve as health science libraries have dutifully addressed their core mandate when supporting a health facility or health training institute. However, they reported to not independently deal with external community to promote health.<br><br>Librarians were very aware of the SDG 3 general goal whoever were mostly unaware of the specific targets.	Librarians are rarely at the forefront of health promotion campaigns.<br><br>The paper notes, “for any success in a wider level of collaboration not only should information professionals possess the skills needed to collaborate with other stakeholders to achieve health goals but also need different channels of communication across a number of different contexts.” -p14
10	Student Wellness & Academic Libraries: Case Studies and Activities for Promoting Health and Success	Duffy, M (2021) [35]	Case study (Book)	n/a	USA	General student population		this book aims to define and discuss wellness and group certain activities in the library as wellness initiatives.	A range of wellbeing initiatives from different universities were described in the book (including digital wellness, therapy dogs, physical activity promotion)	The authors recognise the academic libraries as playing a crucial role in promoting physical health on campus. According to the authors, library spaces are perceived as much safer than a traditional gym environment.	

11	Using robot animal companions in the academic library to mitigate student stress	Edwards, A. (2022) [36]	Quasi-Experimental design	Western Michigan University	Michigan, USA	General student population		This paper investigated the effect of interactions with robot animals within the library on self-reported levels of stress amongst students.	A robot petting zoo was organised in the main library during finals week and data was collected from participants.	The results showed a significant increase in self-reported happiness and relaxation and decrease in acute stress.<br><br>Participants (n=103) reported feeling less tired and bored compared to how they were feeling before petting the robot animal.	The authors report that the long-term use of companion animal robots needs to still be investigated.
12	Brewing tranquil-tea: Supporting student wellness at an academic library	Eldermire, E.R.B. (2022) [61]	Text and Opinion	Floer-Sprecher Vetinary Library, Cornell's College of Medicine	USA	General student population		To investigate the benefits and challenges of giving out free tea to students in the academic library.	Providing tea was observed to be a simple wellness that helped students to reduce anxiety.<br><br>Impact Several students appreciated the initiative. One student reports, "I appreciate the free tea to help with stress, and it really feels like the school cares about the wellbeing of its students with this small act of appreciation." (P9-10)	Students were grateful and it Offered opportunities to connect with library staff. The tea initiative also helped students with stress.<br><br>The librarians saw benefits to their core library function as it opened opportunities to connect with library users.	Shifting baseline expectations of the library user was a challenge.

13	Visualizing the Silent Dialogue about Race: Diversity Outreach in an Academic Library	Everett, S. (2018) [47]	Empirical research	The University of Akron	United States	General Student population		This paper describes the implementation of the Race Card Project Initiative which was aimed at improving multicultural learning and diversity on campus	Race Card Project.	Library was recognised as a safe third space that allowed the campus community to have deep, meaningful conversations on difficult matters.<br>Over 3 years, 450 race cards were collected and analysed.	The incorporation of social media could expand the conversation about race beyond the physical library.
14	Beyond Therapy Dogs: Coordinating Large-Scale Finals Week Activities	Flynn, H. (2017) [28]	Text and Opinion	Michigan State University	USA	General student population		This paper argues that finals week library events that encouraged students to take calming and healthy breaks would create a space that facilitates wellbeing.	Therapy Dogs, Photobooth, Movie night, Yoga, Free Food (Donuts), Blind date with a book, survival kits	Free Food and therapy dog events was highly appreciated and extremely popular amongst students. Once students come in for activities their overall perception of the library is positive.<br><br>For e.g., over 300 survival kits (with chocolates and reminders were given out to students) which has expanded to 500 kits per semester.<br><br>Hurdles included staffing of activities. Planning finals activities takes a lot of trial and error. It often takes many volunteers to support it.	
15	Improving community well-being through collaborative initiatives at a medical library	Funaro, M.C. (2019) [29]	Case Study	Harvey Cushing & John Hay Whitney Medical Library, Yale University	Connecticut, USA	Health care students		To provide effective ways to reduce stress and help improve the well-being of its users (professional s in healthcare and students) by providing mindfulness activities	"Mindfulness on the Medical Campus" initiative was organised.<br>The initiative comprised a mindfulness program and a dedicated space for alleviating stress in health care students.	Community collaboration was crucial, and the process was feedback-driven, and iterative developmental process.<br>Students were pivotal in driving the success of both programs.	Most of the steps that the library took was aimed at improving wellbeing for the individual. A gap they identified was they didn't focus on structural and systemic methods of improving wellbeing.

16	The Hive Mind: supporting community wellbeing in an integrated public and university library	George, M. (2019) [58]	Text and Opinion (Article adapted from a Presentation)	University of Worcester	Worcester, UK	General student population	Community	This article described the Study Happy wellbeing events. It sheds light on its design, delivery and lessons learned in the process of organising it.	Study Happy Programme is a yearlong program of wellbeing events and initiatives to help student study healthier, happier and smarter.	Defining aims and purpose, identifying your audience, using what you already have and knowing the audience were among the important steps highlighted in the paper. The programme organised 40 events and attracted over 1300 attendees.	It was found that most of wellbeing services were not available after hours which is when community members like students come into the library to study.
17	Bring Your Own Story: The Evolution of a Library Program to celebrate Diverse Voices	Gillum, S. (2022) [48]	Qualitative study	Harriet F. Ginsburg Health Sciences Library, University of Central Florida College of Medicine	USA	General student population and LGBTQ issues		To discuss the benefit of diversity-themed lunch and establishment of a diversity corner within the library to promote inclusivity	Bring Your Own Story – This event brought students and staff together to talk about their experiences.<br><br>Bring Your own Story and Human Library Project- “facilitated dialogues to challenge societal prejudices, stigmas and stereotypes in the various topics such as religion, and sexuality” (pg142).	The event was a success. Different voices were heard and attended learned about new experiences.<br>There were 29 signed up attendees for this event.<br><br>“For the library it was a great opportunity to do something that addressed its mission to be a more inclusive space for all its users and its users had an opportunity to be exposed to its other programmes” (p145).	

18	How the University Library is becoming the hub of mental health support- CILIP: The information association	Green, R (2022) [43]	Text and Opinion	Middlesex University	UK	General student population		The article describes how Middlesex university, and its library has responded to mental health issue amongst students.	A wellbeing resource collection within the library and a student success fest where a range of different kinds of support are integrated in engaging ways, providing free personal e-textbooks on mental health and wellbeing. The library also has designated safeguarding officers (DSOs) to support students and staff with mental health issues.		The article suggests that providing wellbeing information and resources students is an area that needs more attention.
19	Supporting student wellbeing and holistic success: A public services approach	Henrich, K. (2020) [37]	Case Study	University of Idaho	Moscow, Idaho, USA	General student population		The purpose of this paper was to showcase the range of non-traditional programs libraries provide that support students' holistic wellbeing.	The wellbeing activities mentioned in the paper included visits from therapy dogs, makerspace featuring 3D printing, scavenger hunt amongst other things.	Students feedback was positive. Must The programs allowed students to feel a sense of ownership over the library, Activities contributed to aspects of wellbeing such as mental health, financial health, food insecurity and sexual health	

20	Biking to Academic Success: A study on a Bike Desk Implementation at an academic library	Hoppenfeld, J. (2019) [54]	Survey and Observational study	Texas A&M University	USA	General student population		This paper explores the connections between physical health and academic achievement and makes recommendations for the implementation of a successful Bike Desk Project	Installation of six stationary Bike Desks	Impact: 152 reported uses of the bike desks from the survey data (p94). 73% of respondents agreed that it increased their probability of studying in the library (p95). More than half of the respondents self-reported that the biking desks increased their academic achievement (p96).<br>Limitations of spaces were recognised (e.g., those who preferred not to use bike desks in the open)<br><br>Overall, this project was clever and successful outreach opportunity brings students in, and experiment with the idea of improving student success through environmental stimulation.	The authors identifies that often projects that involve physical activity promotion are often ignored when thinking about how to engage users of the academic library in new and exciting ways.
21	Owl about That? The use of animals to engage in students with library services	Houghton, R (2019) [38]	Text and Opinion	Middlesex University	UK	General Student Population		The paper describes how owls were used to create a safe space for students to ask questions in an event that showcased the libraries services	#Wise Owls, an activity within a library where students were encouraged to ask questions in a safe friendly place, to ask for help if they needed it and raise awareness on ways that they can reduce stress through the various services.	Student feedback was positive. No measure of whether the event was successful in impacting wellbeing. However, 600 owl badges were handed out each day.	Insufficient feedback was gathered from students.

22	Therapy Dogs in Academic Libraries: A Way to Foster Student Engagement and Mitigate Self-Reported Stress during Finals	Jalongo, M R. (2015) [39]	Case Study	Indiana University of Pennsylvania	Pennsylvania, USA	General student population		This article makes a case for the inclusion of therapy dogs in the library to increase student participation, build a sense of community and reduce stress levels	Therapy Dogs in the library were organised.	The study found that when therapy dogs initiatives are executed well based on evidence of need, it can exert a positive impact and encourage students to use the library more.	
23	Building bridges with book club: Supporting international students' comfort and belonging on campus	Jansen A. (2019) [44]	Case Study	Penn State-Brandywine campus library	Middletown Township, Pennsylvania, USA	General student population (domestic and international student s)		To discuss the effects of a library-run book club for students and how they might encourage feelings of belonging amongst international students.	Book Club with the idea of helping students to better understand experiences of international students	Increased cultural awareness and sharing between domestic and international students. The initiative "helped international students ease into social situations with students outside their cohort" (p582). There was an increase in use of library services by international students. Re-imagining the library as a place for more recreational reading to occur freed students to use it besides when they faced with assignments as well.	
24	Positive effects of mindfulness practices in academic performance and well-being	Karadjova-Kozhuharova, K.G. (2023) [30]	Qualitative study	Georgia Southern University	USA	General student population		The purpose of this study was to explore the positive effects of mindfulness amongst university students.	Brain Booth, an experiential space in the university library with the purpose of reducing stress, optimizing learning, and educating students about the 'mind-body connection', was set up.	The findings showed that Brain Booth was successful, positively influencing their wellbeing as well as overall academic performance. Four areas that brain booth activities helped students included "1) to relax and destress; 2) calm down; 3) foster focus and clarity and 4) actively engage and reenergize" (p879).	

25	Take a Paws: Fostering Student Wellness with a Therapy Dog Program at Your University Library	Lannon, A. (2015) [40]	Case Study	McGill University	Montreal, Canada	General student population		This article reports of therapy dog programs that were carried out in McGill University to reduce stress among students	Therapy dog visits to the main library during exam period. A questionnaire was given to participants to collect data about stress levels before and after interaction with a therapy dog.	Data collected from the project participants (80.7% of which were undergrad students) showed most of them (94.7%) reported reduction in their stress levels.	
26	Student wellness through physical activity promotion in the academic library	Lenstra, N. (2020) [31]	Book chapter (review of literature)	N/a	USA, Canada			This literature review explores how and why physical activity promotion activities are organised in Canadian and American academic libraries.	Range of different activities including installation of treadmill desks in study areas, finals week yoga, and “check out” bicycles.	The paper found that academic librarians supported physical activity through three main categories i.e. “(1) special programs, (2) new uses of spaces, and (3) new collections (p231)”	More research and conversations around making the library a physically active space should be happening.
27	Feasibility of Retrofitting a University Library with Active Workstations to Reduce Sedentary Behavior	Maeda, H. (2014) [55]	Non-randomised experimental study.	University of Wisconsin-Milwaukee	Milwaukee	General student population		To investigate the feasibility of including portable pedal machines as a means of reducing sedentary behaviours in an academic library	11-week intervention where active workstations such as bike desks were fitted in the physical library.	Including portable pedal machines to promote active lifestyle choices seemed feasible, however, its effectiveness needed to be studied and improved further. Uptake was skewed, favouring a certain group of students (male over female).<br>The pedal machines were used at least once by nearly 7% of the student cohort.	Libraries are environments which tend to encourage and/or facilitate sedentary behaviours. However, they remain an understudied environment for sedentary behaviour interventions.

28	Releasing Steam: Stressbusters to Market the Library as Place	Newton, D. (2011) [56]	text and opinion (marketing column)	College of Potsdam	New York	General student population		This paper reported on finals weeks activities that were carried out within the academic library to alleviate stress for students	Stress busters: a range of activities from checkers to mini-golf type were organised during finals week	The finals week activity reportedly students feeling more positive towards both their work and the academic library.	
29	HIV/ AIDS information promotion at the library: creative campaigns for young adults	Norton, H.F. (2019) [45]	Case Study	University of Florida (UF)	Florida, USA	General student population	Community	The paper describes a project i.e., Creative Campaigns Project which to enhance access of HIV/AIDS information materials amongst UF students and to improve information seeking behaviours trough training of health care providers.	Creative Campaigns project included three components- a graphic novel contest, social media promotion, and training for health care providers,	A few quality submissions for the creative campaigns project were received.<br><br>Social media campaign generated interest in the library's initiative. Campaign posts had more engagement than other posts. The links to the graphic novel contest was clicked 297 times (p228)<br><br>The training program highlighted resources that health professionals were unaware of.<br><br>The project expanded librarians' view about how to generate awareness about health topics through creative campaigns.	One limitation that was identified in the project was that impact of the campaign on the UF students could not be measured. Also, there was no explicit evaluation of the how the awareness campaign impacted health behaviours.

30	Community outreach and engagement in the time of COVID-19: Efforts & approaches of academic libraries	Peñaflor, J. (2021) [32]	Survey	Multi-University (23 universities)	South-east Asia i.e. Brunei, Darussalam, Indonesia, Cambodia, Laos, Malaysia, Myanmar, Singapore, Thailand, and Vietnam	General student population	Community	The purpose of this study was to examine the range of community engagement and outreach activities organised by university libraries during the COVID-19 pandemic when the physical library was operating at very limited capacity.	A survey was conducted on the following specific categories of outreach: "Collection-based outreach, instruction and services-based outreach, whole person outreach, just for fun outreach, partnerships and community-focused outreach and multi-pronged themed events and programming" (p1). Whole person outreach initiatives were aimed at helping people at an individual level. It included an example of Fit and Firm activity weekly, a suicide prevention campaign, meditation and dance lessons and a project related to women empowerment	An estimated 69.57% of academic libraries offered outreach programs that belonged to the "whole person" and "partnerships and community-focused outreach" categories<br><br>"Libraries are innovative and tend to deliver their services in innovative ways. With the increase in remote services and programs, libraries are also turning their focus towards coming together as a community" (p38)<br><br>For librarians, the project helped expand how they could use raise awareness about health literacy in creative ways.	There is a need to turn outward into the community and maximise partnerships.

									(Page 36)<br><br>Community focused outreach involved participating in civic activities. Examples of initiatives include collaboration with an elderly club to promote reading, donation of books, environmental awareness drives, etc		

31	Are Canadian medicine librarians directly supporting medical student health and wellness? A nation-wide survey	Phinney, J (2021) [59]	Survey (Nation-wide)	Canadian Universities	Canada	General student population		The aim of this study was to investigate whether and how Canadian health science librarians are supporting medical student wellbeing while at medical school.	An electronic survey offered in two languages was circulated among librarians in two Canadian health science libraries and they were asked to reflect on a range of different wellness initiatives (e.g. support for distressed students, wellness-themed displays or spaces.	Data found that undergraduate medical librarians are taking steps to provide wellbeing support to their student users. A lot of respondents fourteen out of seventeen indicate that they had been participated in wellbeing activities to different extents.<br>More than half made teaching modifications and nearly half made purchases to help with students' wellness and wellbeing. However, when it came to engagement or promotion activities such as organising wellness themed displays or spaces withing the library, many respondents said No.	The authors found that papers the described wellbeing interventions in libraries failed to settle on a core concept or definition of wellbeing and so for librarians who might lack the expertise to support wellbeing initiatives (p171).<br><br>Another gap and area of research interest could be examining librarians' willingness and comfort in supporting the wellness of its users.
32	Festival of wellbeing at Teeside University	Porritt, F. (2019) [33]	Text and Opinion	Teesside University	UK	General Student Population		This paper describes a yearly wellbeing Initiative with a range of activities that enable students to "see a continuum between study and other activities" pg. 16	Festival of Wellbeing (Included activities such as Tai Chi, Yoga, and relaxation, talks that linked nutrition and mental health, sports, and a reading for pleasure activity	The session was reported to be very popular among students.	

33	Finding the Finals Fairy: Outreach and stress during finals	Prichard, A. (2020) [57]	Case study, Essay	Salisbury University	USA	General student population		The paper describes efforts in a university to alleviate stress during finals	Hide and seek treasure hunt game with prizes was organised with the intention of helping students destress.	The initiative found student engaging with the activity as a fun break from their study. The activity also facilitated students' discovery of spaces within the library which normally they wouldn't explore.	
34	Studying for Exams Just Got More Relaxing – Animal-Assisted Activities at the University of Connecticut library	Reynolds, J A (2011) [41]	Text and Opinion	The University of Connecticut	Connecticut, USA	General student population		The study documents how animal assisted activities can be very beneficial to students' wellbeing during the final's week.	The study describes the Paws To Relax initiative- a therapy dog program.	Student interaction was very positive, and students took time out of their day to appreciate what it did to reduce their stress.<br>The initiative is also a welcome relief for staff since they get a break from their day.	
35	Supporting Student Wellness: De-stressing Initiatives at Memorial University Libraries	Rose, C. (2015) [34]	Survey	University of Newfoundland	Newfoundland, Canada	General student population		This article looks at the connections between mental health and academic performance and how stress anxiety and depression affect the latter.	Initiatives that were organised for improving student wellbeing included extended hours; Breathe, a yoga and mindfulness program; pet therapy; serving free beverages and snacks to students; and organising micro-breaks (p1)	Students self-reported that the range of wellness initiatives offered by the library helped with reducing stress.<br>Out of the 194 attendees who completed the questionnaire, 89% agreed that the program helped them de-stress.<br><br>Although an unintended consequence, these wellbeing initiatives promoted academic libraries to the wider community.	A gap that was identified was that most of the research is targeted at undergrad students. Data related to graduate students seem to be an area for further study. In addition, how much of the wellbeing services should be a part of the business-as-usual library services is another potential area for research.

36	A Display of Tolerance: Partnering with Multicultural Groups to Mount a Juried Art Exhibition	Seymour, C. (2012) [60]	Text and Opinion	Union College	USA	LGBTQIA students	Community	Event was motivated by concerns about hate crimes against the LGBTQIA community	Art Exhibition	The exhibition helped further a dialogue about LGBTQIA issues.<br>Awareness about LGBTQIA issues promoted acceptance on campus and amongst community members.<br><br>“The idea of bringing students, faculty, and staff together for meaningful social exchange seemed like a fitting approach to this community issue- P1<br><br>Impact:<br>Entries in our inaugural exhibition’s guestbook indicate that the conversation our installation was meant to inspire was indeed an ongoing one”- p3	Opportunities for reflection in such an event; Need for more partners to support the library.
37	Academic Library Service for Disables Students: Today and Tomorrow	Stone, E. W. (1983) [51]	Text and opinion	Catholic University	USA	Students with Disability		To focus on the status of disabled students, services available to them, and make recommendation for academic libraries serving disabled students	Orientation session and Interview of Disabled students and personalisation of services according to their disability and reimagining of library spaces. Catholic university has a special room for handicapped individuals which serves as a private space to use library equipment without any disturbance (p16-17)	A quality library service that is intentionally aimed at increasing participation, integration and involvement of disabled students improves outcomes for students and uptake of library services. P. 27	The author has highlighted that not enough has been done in academic libraries that allow students with disability to belong to spaces and act with agency.

38	Senior CHAT: a model for health literacy instruction	Strong, Mary Lou (2012) [52]	Case Study	South-eastern Louisiana University	Louisiana, USA		Community (Senior residents in Louisiana)	The aim of the project was to promote health literacy and eventually overall health and wellbeing among senior members in the Louisiana parish through basic computer skills program	Senior CHAT is a Consumer Health Awareness Training program that aims to improve health literacy through basic computer instruction	The program in South-eastern University was promoted 'lifelong learning' in Louisiana; established new community partnerships; and also helped senior citizens to be able to confidently find health information.<br><br>53.8% of the study participants reported that they were able to confidently find health information they needed compared to pre-program levels	A huge gap in addressing seniors' health concerns and their basic need to find authoritative health information.
39	Combining efforts: Libraries as mental health safe spaces	Thomas, S. (2019) [49]	Text and Opinion	Marshall University Libraries	USA	General student population	Community	The goal of this project was to encourage library users (both students and staff) as well as faculty to create art that was informed by and helped with their mental health	MU Universities mental health initiative began with an art exhibition called "Don't call me crazy: Resilience through art".<br><br>This mental health initiative was expanded to include 3 components. Resilience through art, resilience through community and resilience through education.	Various wellness initiatives run by the library for mental health highlighted how library is a safe space where users can seek answers to their concerns without shame and with protected anonymity.<br><br>18 individuals reportedly attend the session on anxiety and depression.<br>The webpage linking to resources for community also reported nearly 2000 visits between 2018 and 2019.	

40	Supporting Student Wellbeing in the University Library: A core Service or a Distraction	Walton, G. (2018) [5]	Text and Opinion	Sector-Wide within UK	UK	General student population		The aim of the paper was to showcase wellbeing initiatives in the sector and provide recommendations to the sector regarding the same.	The range of services highlighted in the paper fall in 4 categories- physical space (e.g. Meditation room), digital support (digital wellbeing resources), collection (e.g. wellness books) provision and other (e.g. Animal assisted activities)	The authors recognise that since it is perceived as a place of safety and is trusted amongst its users, the academic library is positioned well to provide wellbeing support	The authors recognise that there is a strong need and impetus for academic libraries to participate in a strategic response to improve student wellbeing which currently is absent in most universities.
